# Hybrid geostatistical and deep learning framework for geochemical characterization in historical mine tailings

**DOI:** 10.1038/s41598-025-19441-5

**Published:** 2025-10-07

**Authors:** Keyumars Anvari, Jörg Benndorf, Gabriel Gerber, Uta Alisch

**Affiliations:** 1 Faculty of Geosciences, Geoengineering and Mining, Institute of Mine Surveying and Geodesy, TU Bergakademie Freiberg, 09599 Freiberg, Germany; 2 GICON Resources GmbH, 01219 Dresden, Germany

**Keywords:** Mine tailings, Geostatistics, CNN–RNN, Geochemical modeling, Resource recovery, Circular economy, Geochemistry, Engineering, Mathematics and computing, Environmental impact

## Abstract

Sustainable mine tailings management has become a worldwide priority given increasing critical raw materials (CRMs) demand and growing environmental concerns. While these anthropogenic deposits are often enriched with useful metals, they may also contain hazardous substances and thus provide both opportunities for resource recovery and environmental risk. In this work a hybrid geostatistical–deep learning framework was established to model geochemical distribution in old tailings. This study integrates ordinary kriging (OK) with a one-dimensional convolutional neural network and a bidirectional long short-term memory model (1D CNN and BiLSTM). The hybrid relies exclusively on features derived from the OK spatial covariance structure, computed from covariance matrices over the sampled locations, to inform the deep model and enhance prediction accuracy. The framework, applied to a historical tailings site, significantly outperformed traditional geostatistical methods as it can provide high-resolution predictions across all points of interest, while accounting for spatial heterogeneity. These results highlight the applicability of this strategy in sustainable resource recovery and environmental remediation, in accordance with circular economy concepts.

**Supplementary Information:**

The online version contains supplementary material available at 10.1038/s41598-025-19441-5.

## Introduction

The global demand for critical raw materials (CRMs), including cobalt, lithium, rare-earth elements and key metals such as copper, gold, zinc and aluminum, keeps rising in response to population growth, clean-energy deployment and rapid technological innovation^1,2^. Scaling up extraction creates larger volumes of fine-grained mine tailings that still contain hazardous metals, such as copper, zinc, nickel, arsenic, and lead, and sulfide minerals^3^. Once exposed to oxygen and water these sulfides oxidize, producing acid mine drainage (AMD) with low pH and high metal solubility that degrades soils, groundwater and aquatic ecosystems^4,5^. Many studies have identified ecological risks from mine tailings including the release of toxic elements and the modification of hydrological and geochemical processes^3,6–10^.

Economic incentives and tighter regulation now promote tailings re-mining, reframing these deposits as secondary raw materials with real market value^11^. Historical processing inefficiencies left substantial residual metal inventories^12–15^so modern recovery can extract CRMs while lowering long-term liabilities. Reprocessed tailings already substitute virgin feedstocks in construction products such as ceramics, cement and bricks^11,16,17^, and mafic or ultramafic residues offer reactive surfaces that lock CO₂ as stable carbonates^18^. Coupling remediation with CRM recovery therefore mitigates environmental harm, strengthens resource security and supports sustainable mining.

Achieving these outcomes depend on a detailed knowledge of the spatial distribution, composition and heterogeneity of mine tailings. Recent studies have explored the feasibility of recovering metals^12^ and the chemical, mineralogical, and textural characterization of tailings^14^. Meanwhile, geostatistical and spatial modeling approaches have become indispensable for capturing the inherent variability of tailings deposits, incorporating inverse distance weighting^19^ and combined inverse distance weighting-kriging approaches^20^ to kriging-based modeling of elemental attributes^21^, leave-one-out cross-validation^22^, and advanced methods like transitive covariogram and kriging^23^, co-kriging^24^, compositional data analysis (CoDa)^25^, and universal kriging-based sequential Gaussian simulation^13,26^. These methods have steadily refined our capacity to characterize complex mineral wastes and support sustainable tailings management.

Although these advances are promising, classical geostatistical methods struggle with the complexity of tailings spatial structures. The geometrical complexity, heterogeneity and poor continuity of mine tailings makes accurate resource estimation challenging^23^. Although ordinary kriging (OK) can become computationally demanding for very large datasets^27,28^, recent advances, such as covariance‑tapering^29^, sparse‑matrix solvers^30^, moving‑window/local kriging^31^ and cluster‑based parallelization^32^, now let OK handle millions of points efficiently. Nevertheless, because OK is the linear minimum‑variance estimator it yields relatively smooth maps, which can attenuate local extremes and thus underestimate high values while overestimating low values^33^.

Meanwhile, rapid improvements in computing power and data analytics have led to the emergence of artificial intelligence (AI) and deep learning (DL) approaches that address data complexities with far more flexibility. AI, defined as the development of computer systems capable of tasks typically requiring human intelligence, such as reasoning, learning, and language comprehension, has already found widespread application across diverse fields^34^. From computer vision^35–37^ and medicine^38–40^ to geology^41–46^ and porous media^47–49^. Methods based on AI have shown impressive results. One; the mining industry has reaped a huge benefit from AI. Now combine this with the existence of real-time sensors generating large datasets that are well suited for data-driven methods to improve decisions in mining operation^42,50,51^.

However, caution is crucial for the AI integration in geosciences. Advanced DL architectures require substantial representative data for training and must also meet physics-based constraints to avoid solutions that violate the fundamentals^27^. Geological principles, such as spatial continuity, can be woven into model architectures or loss functions to enforce scientific consistency^27,52–54^. In fact, convolutional neural networks (CNNs) and recurrent neural networks (RNNs), especially long short-term memory (LSTM) variants, are increasingly employed to capture spatial and temporal features in geological datasets^27,52^. CNNs work well at identifying localized spatial features; thus, they can be well-suited to analyzing geospatial imagery^55^. RNNs, specifically long short-term memory (LSTM) variants, are particularly adept at capturing sequential dependencies, providing some utility in time-varying and spatially complex datasets^56^. Notably, hybrid CNN–RNN approaches combine these functions to exploit both spatial and temporal features, e.g., for land cover change detection from multitemporal satellite imagery^57^.

While CNNs alone may overlook sequential dependencies and RNNs may struggle with spatial features, a combined CNN–RNN approach unites these complementary strengths. Studies have shown that this fusion enhances predictive accuracy in domains spanning rainfall-runoff modeling, music classification, and beyond^58–60^. By jointly encoding localized spatial variability and temporal relationships, hybrid architectures deliver a more robust and comprehensive interpretation of geoscientific phenomena^59,61^​​.

In this paper, we present an innovative geostatistical CNN–RNN (GCNN–RNN) framework for modeling the geochemical composition of historical mine tailings. The approach we propose integrates kriging-derived spatial covariance matrices and variogram parameters, thereby incorporating local spatial relationships, into a 1D CNN–bidirectional LSTM network that models complex, nonlinear dependencies. Careful implementation of callbacks and systematic residual checks ensures both numerical stability and geological consistency in the final outputs.

This innovative approach enhances the precision of geochemical modeling, addressing the challenges posed by the tailings’ heterogeneous and anthropogenic nature. By merging spatial correlation structures with the pattern-recognition strengths of deep networks, our framework achieves enhanced predictive accuracy for tailings re-mining applications. We validate this approach on a historical tailing deposit, demonstrating its practical utility for geochemical resource assessment and its potential to support more sustainable and efficient mining practices.

## Materials and methods

### Methods

**Fig. 1:**
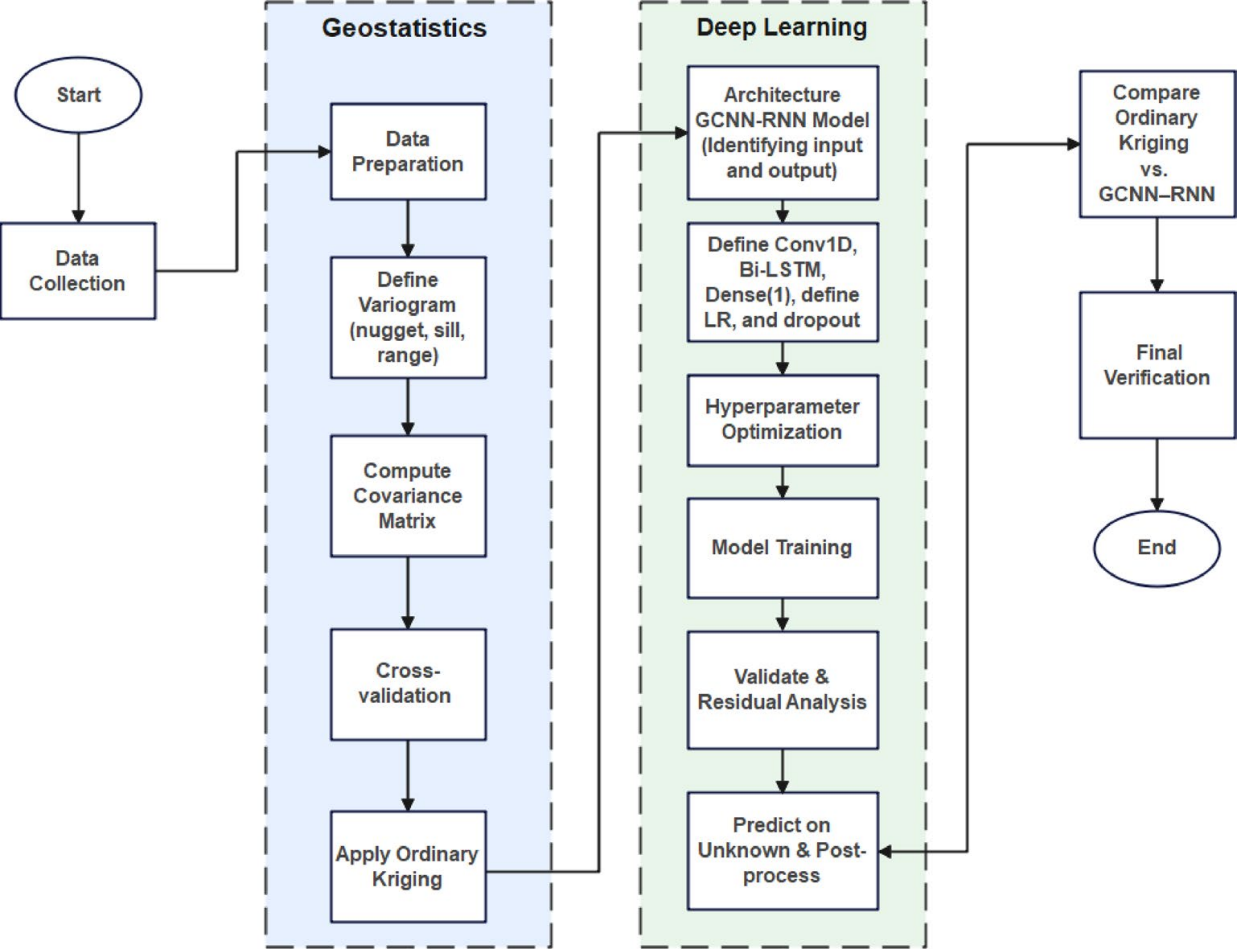
Workflow for hybrid geostatistical and deep learning approach (GCNN–RNN) applied to geochemical variable prediction.

As outlined in Fig. 1, the hybrid GCNN–RNN approach begins with spatial data preparation, including downloading, cleaning, and annotating geochemical samples to separate known from unknown locations. An experimental variogram is fitted to quantify spatial dependence, and its parameters (nugget, sill, range) are selected by cross-validation to minimize prediction error. The fitted model defines the spatial covariance function used to compute the covariance matrix among samples. From this matrix, a compact descriptor of local spatial continuity is calculated for every location as the row-wise average covariance. OK is then run to produce a conventional baseline and corresponding spatial maps for comparison.

The deep learning component is configured with a 1D convolutional neural network–bidirectional long short-term memory (CNN–BiLSTM) architecture, sequentially structured as Conv1D → BiLSTM → Dense(1). Critical hyperparameters, such as learning rate and dropout rates, are optimized accordingly. A dedicated hyperparameter tuning step precedes model training to ensure optimal configuration. Training is carefully managed using specialized callbacks, including EarlyStopping, ReduceLROnPlateau, and ModelCheckpoint, to ensure stable convergence and optimal model performance.

In the final steps, model residuals undergo rigorous validation and analysis. Predictions for previously unseen spatial locations are generated and post-processed. Finally, the performance of the hybrid GCNN–RNN model is compared against the OK approach, providing a comprehensive verification that confirms the model’s capability to deliver robust geochemical predictions and high-resolution spatial characterizations suitable for mine tailings assessment.

#### Geostatistical framework

The study employs geostatistical techniques to characterize spatial variation in geochemical elements through OK. Spatial dependence is first quantified with an experimental variogram that relates semi-variance to lag distance, and among the standard analytical forms (exponential, Gaussian, spherical) the spherical model is adopted because it reaches a finite sill and is widely recommended in environmental geochemistry. Its three parameters (nugget, sill, range) respectively describe micro-scale variance, total spatial variance and the correlation distance, and they subsequently define the covariance function required for kriging^62,63^.

Assuming second‑order stationarity (a constant mean m and a covariance that depends only on the separation vector h), the semi‑variogram γ(h) and the covariance function C(h) are linked by

1 $$\gamma \left( h \right){\text{ }}={\text{ }}C\left( 0 \right)~ - ~C\left( h \right)$$

Cross-validation is employed to refine the variogram parameters $\:\left(n,s,a\right)$ for each geochemical, guiding parameter selection toward minimal prediction error and thereby enhancing the accuracy of spatial predictions. The procedure follows these steps:

1. Parameter Grid Search: A range of plausible values for $\:n$, $\:s$, and $\:a$ is defined. Each triplet {$\:\left(n,s,a\right)$}c is tested systematically. Plausible bounds for the nugget (n), sill (s) and range (a) were centered on rapid anchor estimates *(n*_*₀*_, *s*_*₀*_, *a*_*₀*_*)* taken from the experimental variogram. We allowed *n* to vary from 0 to 0.5 *s*_*₀*_, *s* from 0.5 *s*_*₀*_ to 2 *s*_*₀*_ and *a* from 0.25 *a*_*₀*_ to 1.25 *a*_*₀*_.
2. Leave-one-out estimation: for each candidate triple.
  Remove a single observation $\:{x}_{i}$ (with the observed value $\:z\left({x}_{i}\right))$ from the dataset.
  Use the remaining observations to predict the value at $\:{x}_{i}$.
  Compute the squared error $\:{\left[{z}^{*}\left({x}_{i}\right)-z\left({x}_{i}\right)\right]}^{2}$.
  Repeat for all $\:i=\text{1,2},\dots\:,N$ samples and average the errors.
3. Selection: Choose the parameter triple$\left( {n_{{best}} ,s_{{best}} ,a_{{best}} } \right)$ that minimizes the mean squared cross-validation error.

Mathematically, let $\:\varvec{Z}=\left\{z\left({x}_{l}\right),z\left({x}_{2}\right),\dots\:,z\left({x}_{N}\right)\right\}$ represent the observed values. For each parameter triplet $\:\left(n,s,a\right)$, the cross-validation score is:

2 $$CV\left( {n,s,a} \right)=\frac{1}{N}\mathop \sum \limits_{{i=1}}^{N} {\left[ {z_{{ - i}}^{*}\left( {{x_i}} \right) - z\left( {{x_i}} \right)} \right]^2}$$

where $\:{z}_{-i}^{*}\left({x}_{i}\right)$ denotes the kriging estimate of $\:z\left({x}_{i}\right)\:$computed with the $\:i$-th observation excluded. The optimal parameters satisfy by Eq. (3):

3 $$\left( {{n_{best}},{s_{best}},{a_{best}}} \right)~=~\arg ~\hbox{min} CV\left( {n,s,a} \right)$$

This grid search-based optimization ensures robust parameter estimation, enhancing the reliability of subsequent spatial predictions.

This grid-search optimization ensures robust parameter estimation, enhancing the reliability of subsequent spatial predictions. Using the optimized variogram, OK then provides best-linear-unbiased estimates of element concentrations at unsampled locations while delivering kriging variance as a quantitative measure of prediction uncertainty. OK is preferred here because it avoids imposing a global mean (simple kriging) or an explicit trend model (universal kriging) yet fully exploits the spatial covariance structure derived from the fitted spherical model, ensuring scientifically coherent and conceptually continuous spatial prediction.

#### Hybrid GCNN-RNN model

The hybrid GCNN–RNN approach leverages geostatistical insights (i.e., OK-derived features) alongside a deep neural network composed of convolutional and recurrent layers. The resulting GCNN–RNN architecture combines the spatially informed features from geostatistics with advanced sequence modeling.

Traditional deep learning models may not fully capture spatial correlation structures inherent in geostatistical data when relying solely on raw input features (e.g., observed concentrations, spatial coordinates)^64^. To address this, the proposed approach integrates kriging-based information, specifically the spatial covariance derived from the fitted variogram. Concretely, each sample’s feature vector includes (1) the observed concentration, (2) its spatial coordinates (*X*,* Y*), and (3) an average covariance measure (the mean of the sample’s row in the covariance matrix). By appending this geostatistical feature, the subsequent CNN–RNN subnetwork can learn nonlinear, spatially dependent patterns more effectively. The kriging-based feature offers domain-specific knowledge of local structure, while the CNN and Bi-LSTM layers provide powerful representation learning and sequence modeling capabilities, leading to refined predictions that surpass those of simple kriging alone.

Although OK alone performs modestly in this dataset due to its tendency to smooth out local extremes, it still captures essential spatial patterns through its covariance structure. In our hybrid model, we do not rely on kriging’s point estimates as predictive inputs. Instead, we extract the spatial covariance derived from the variogram and use it as one of four input features to inform the network about local spatial continuity. This kriging-derived feature serves as a spatial context cue and is processed alongside observed concentrations and coordinates. The model is designed with mechanisms such as dropout, regularization, and early stopping that allow it to learn when and how much to trust this information.

**Fig. 2:**
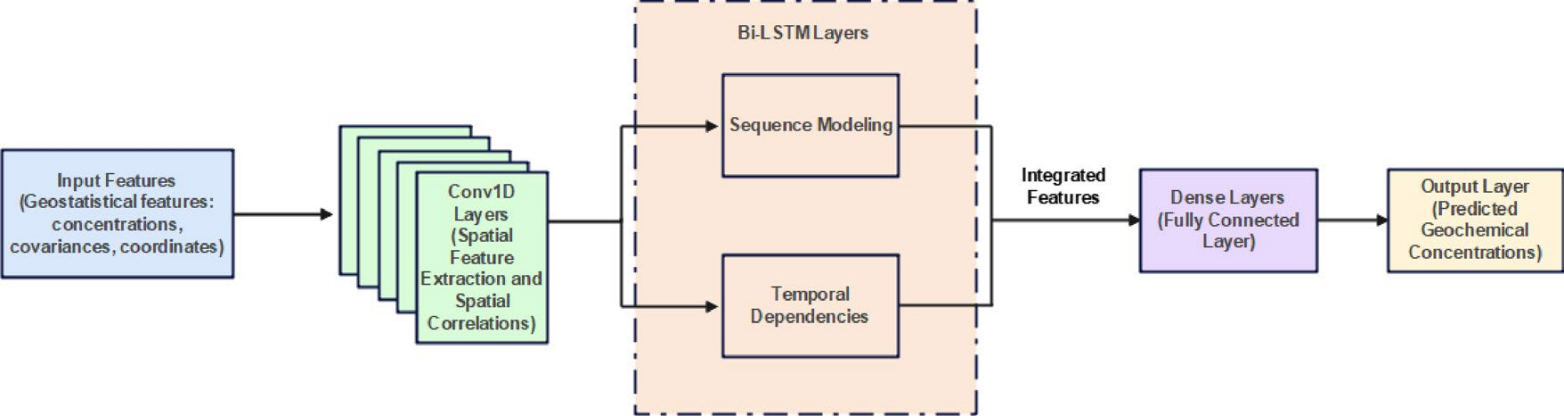
Architecture of the hybrid GCNN–RNN model for geochemical concentration prediction.

The GCNN–RNN architecture (Fig. 2) builds upon a 1D Convolutional Neural Network (CNN) to extract feature representations, followed by a Bidirectional LSTM (Bi-LSTM) layer that captures temporal or sequential dependencies (here used in a “sequence-of-features” sense).

Formally, let X∈R^*B*×*T*×*d*^ denote the input tensor, where:

- *B* is the batch size,
- *T* is the time or sequence length (in this implementation, *T* = 1 since we treat each input row as a “1-timestep” sequence),
- *d* is the dimensionality of each feature vector (e.g., concentrations, coordinates, and kriging-based covariance).

1. Convolutional Layer (Conv1D):
  A 1D convolution with kernel size *k* and filter size *F* is applied to each feature vector:
  4 $${H^{\left( {conv} \right)}}=\sigma \left( {{W_{conv}}*X+{b_{conv}}} \right)$$
  where σ(⋅) is a nonlinear activation function (here, LeakyReLU), $\:{W}_{conv}$ and $\:{b}_{conv}$ are trainable parameters, and $\:*$ denotes the convolution operation across the last dimension (i.e., along the feature dimension *d*).
2. Bidirectional LSTM (Bi-LSTM): The output of the convolutional layer is then passed to a bidirectional LSTM. A single LSTM cell computes, at each step *t*:
  5 $$\begin{aligned} \dot{I}_{t} & = \sigma \left( {W_{i} x_{t} + U_{i} h_{{t - 1}} + b_{i} } \right),f_{t} = \sigma \left( {W_{f} x_{t} + U_{f} h_{{t - 1}} + b_{f} } \right), \\ o_{t} & = \sigma \left( {w_{o} x_{t} + U_{o} h_{{t - 1}} + b_{o} } \right),\tilde{c}_{t} = \tanh \left( {W_{C} x_{t} + U_{C} h_{{t - 1}} + b_{c} } \right), \\ c_{t} & = f_{t} \odot c_{{t - 1}} + i_{t} \odot \tilde{c}_{t} ,~ \\ h_{t} & = o_{t} \odot \tanh \left( {c_{t} } \right) \\ \end{aligned}$$
  where $\:{\dot{I}}_{t},\:{f}_{t},\:{o}_{t}\:$are the input, forget, and output gates, respectively; $\:{\text{c}}_{t}\:$is the cell state; $\:{h}_{t}$ is the hidden state; and ⊙ denotes element-wise multiplication. A bidirectional variant processes the sequence in both forward and backward directions, concatenating hidden states to form the final output.
3. Dense Layers:
  Following the Bi-LSTM output (a vector of dimension 2×hidden units due to bidirectionality):
  6 $$z=\varphi \left( {{W_d}{h_{Bi - LSTM}}+~{b_d}} \right)$$
  where $\:\phi\:$(⋅) is often ReLU. Finally, a single output neuron with a linear activation estimates the target concentration:
  7 $$\hat {y}=w_{o}^{ \top }z+{b_o}$$
4. Regularization and Optimizer:
  Dropout is applied after convolution and LSTM layers to reduce overfitting.
  *L*_2_ Regularization is incorporated into convolutional and LSTM kernels via $\lambda \left\| {W_{2}^{2} } \right\|$.
  Optimizer: using the Adam optimizer with a reduced learning rate (10^−4^).

Input features

Unlike standard deep learning approaches that rely solely on the observed concentration values, the GCNN-RNN model integrates:

1. Concentration (ppm): The observed (for known points) geochemical concentration of the target element.
2. Spatial Coordinates (*X*,* Y*): The two-dimensional location of each sample, facilitating the model’s spatial awareness.
3. Average Covariance: Obtained by averaging the kriging covariance matrix row for each sample (i.e., the mean of spatial covariances between that location and all other points). Let *C*∈R^*N*×*N*^ be the covariance matrix; the “average covariance” for sample *i* is:

8 $$A\nu gCo{v_i}=\frac{1}{N}\mathop \sum \limits_{{j=1}}^{N} {C_{i\dot {j}}}$$

Therefore, each sample’s input vector $\:{x}_{i}$ can be represented as:

9 $${x_i}=\left[ {Con{c_i},{X_i},{Y_i},AvgCo{v_i}} \right]$$

When reshaped for the convolutional layer, each batch of inputs becomes *X∈R*^*B×1×4*^. The dimension “1” corresponds to a single “time step,” ensuring that CNN–RNN layers can be applied seamlessly.

We selected average covariance for integration into the deep learning model based on its physical interpretability and strong empirical performance within our dataset.

Training

Data preparation

1. Known vs. Unknown Partition:
  Known data {(*X*_*i*_,*Y*_*i*_,*z*_*i*_)} are measured points in the field.
  Unknown data {(*X*_*j*_,*Y*_*j*_, $\:{z}_{j}^{*}$)} are locations where the concentration $\:{z}_{j}^{*}$ was initially approximated by OK.
2. Feature Assembly:
  Compute the covariance matrix C using the spherical variogram.
  Derive “average covariance” for each known and unknown location.
  Concatenate [*Conc*,*X*,*Y*,*AvgCov*] into a final feature matrix.

Hyperparameter tuning

- EarlyStopping: Monitors validation loss and halts training if no improvement is seen after 50 epochs (patience).
- ReduceLROnPlateau: Reduces the learning rate by a factor of 0.5 when validation loss stagnates for 25 epochs.
- ModelCheckpoint: Saves the weights of the best-performing epoch to prevent overfitting.
- Batch Size and Epochs: A batch size of 8 is used, and training can run up to 1000 epochs (often stopped earlier by EarlyStopping).

Let θ denote all the trainable parameters (weights and biases). The model’s objective is to minimize the MSE on training data:

10 $$\mathcal{L}\left( \theta \right)=\frac{1}{M}\mathop \sum \limits_{{i=1}}^{M} {\left( {{{\hat {y}}_i}\left( \theta \right) - {y_j}} \right)^2}$$

where $\:{\widehat{y}}_{i}\left(\theta\:\right)$ is the model’s prediction for sample *i*, and $\:M$ is the mini-batch size (or total number of samples in one epoch, depending on the chosen scheme).

As summarized in the workflow of Hybrid GCNN–RNN approach using the steps outlined in Algorithm 1 below.

**Algorithm 1:**
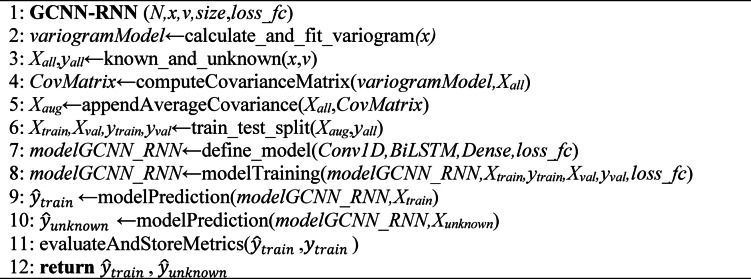
Pseudo-code of GCNN-RNN.

Evaluation metrics

The model’s performance is assessed on both the training set and a held-out validation set using standard regression metrics^65,66^:

1. MSE:

11 $$MSE=\frac{1}{N}\mathop \sum \limits_{{i=1}}^{N} {\left( {{{\hat {y}}_{\dot {l}}} - {y_i}} \right)^2}$$

2. Mean Absolute Error (MAE):

12 $$MAE=\frac{1}{N}\mathop \sum \limits_{{i=1}}^{N} \left| {{{\hat {y}}_i} - {y_i}} \right|$$

3. Root Mean Squared Error (RMSE):

13 $$RMSE=\sqrt {\frac{1}{N}\mathop \sum \limits_{{i=1}}^{N} {{\left( {{{\hat {y}}_i} - {{\hat {y}}_i}} \right)}^2}}$$

4. Coefficient of Determination ($\:{R}^{2}$):

14 $${R^2}=1 - \frac{{\mathop \sum \nolimits_{{i=1}}^{N} {{\left( {{{\hat {y}}_i} - {y_i}} \right)}^2}}}{{\mathop \sum \nolimits_{{i=1}}^{N} {{\left( {{y_i} - \bar {y}} \right)}^2}}}$$

where $\:\stackrel{-}{y}$ is the mean of the observed target values $\:{y}_{i}$​.

These metrics collectively offer insights into both the magnitude of the error (via MSE, MAE, RMSE) and the proportion of variance explained by the model ($\:{R}^{2}$).

The GCNN–RNN framework requires more computational resources than classical geostatistics. All model training and inference in this study were performed on a standard laptop (Intel Core i9-13900 H, 16 GB RAM), demonstrating that moderate-sized datasets can be processed without specialized hardware. However, for substantially larger datasets, practitioners may require a dedicated GPU or cloud computing to ensure practical runtimes.

### Data description

This study explores a synthetic mining dataset representing a large, historical mine tailings deposit. To capture its inherent heterogeneity, 82 drillholes were drilled at depths of 0.3 m to 1.0 m, guided by observed stratigraphic transitions, yielding an effective density of 6.7 points km⁻². The boreholes are spatially distributed across the site to proportionally sample the major facies, ensuring adequate coverage of internal variability for geostatistical modeling. The investigation concentrated on four geochemical elements: zinc (Zn), calcium (Ca), copper (Cu), and sulfur (S), which each element was measured in parts per million (ppm).

**Fig. 3:**
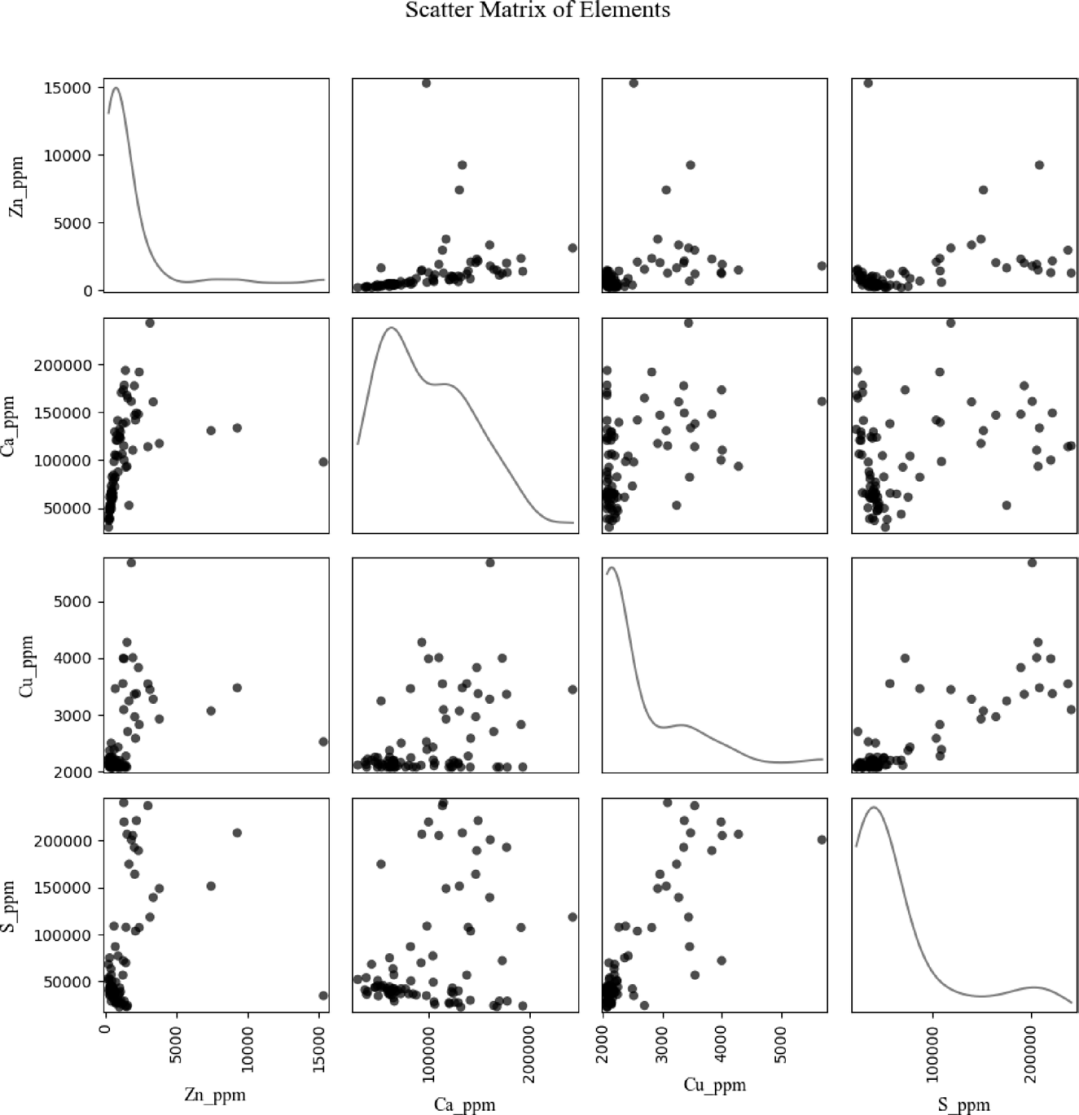
Pair plot of geochemical element distributions and relationships in tailings sample.

A pair plot (Fig. 3) illustrates the interrelationships among Zn, Ca, Cu, and S concentrations, with off-diagonal scatter plots revealing bivariate patterns and diagonal plots showing individual kernel density estimates. Exploratory data analysis of the full dataset and of the training/validation partitions is provided in Supplementary Figures S1–S2. Ca and S reveal strong right-skewed distributions, reaching extremely high concentrations (~ 200,000 ppm), suggesting heterogeneous geochemical behavior^67,68^. Zn concentrations (~ 0–15,000 ppm) are lower but exhibit a weak positive association with S and a scattered pattern relative to Ca. Cu concentrations (~ 2,000–5,000 ppm) are relatively consistent across samples, showing minimal correlation with the other elements. The density plots highlight that most Zn and Cu values cluster toward lower concentrations, while Ca and S have broader, less uniform distributions.

The four analytes collectively flag both environmental hazard and economic opportunity in sulfide-rich tailings. Sulfur is a proxy for pyrite and other acid-generating sulfides; S-rich zones therefore mark areas prone to AMD and elevated metal mobility^69,70^. Ca is chiefly hosted in carbonate or silicate gangue that can buffer acidity, so Ca-rich material can serve as an in-situ neutralizing agent or as cementitious feedstock^71,72^. Zn and Cu occur in historical tailings at residual grades that remain economically attractive for recovery, making them prime targets for re-mining^73–75^. Consequently, mapping the joint spatial distribution of Zn, Cu, S and Ca supports pollutant identification by delineating acid-generating hot spots and metal-leaching plumes while also guiding resource-recovery strategies that focus on Cu-rich blocks and use Ca-bearing material for onsite neutralization or construction applications.

## Results

### Geostatistical framework

The geostatistical analysis for each element was conducted using variogram modeling to characterize spatial dependence. Variogram parameters, including the nugget effect, sill, and range, were derived through a rigorous cross-validation process and are summarized in Table 1. These parameters provided an optimal fit for the spatial interpolation of element concentrations. The cross-validation metrics confirmed that the estimation errors remained within acceptable limits, indicating the robustness and accuracy of the chosen variogram models.

**Table 1:** Variogram parameters derived from cross-validation.

Elements	Nugget	Sill	Range
Ca	0.5	0.6	50.0
Cu	0.4	0.5	50.0
S	0.4	0.5	50.0
Zn	0.5	0.6	50.0

**Fig. 4:**
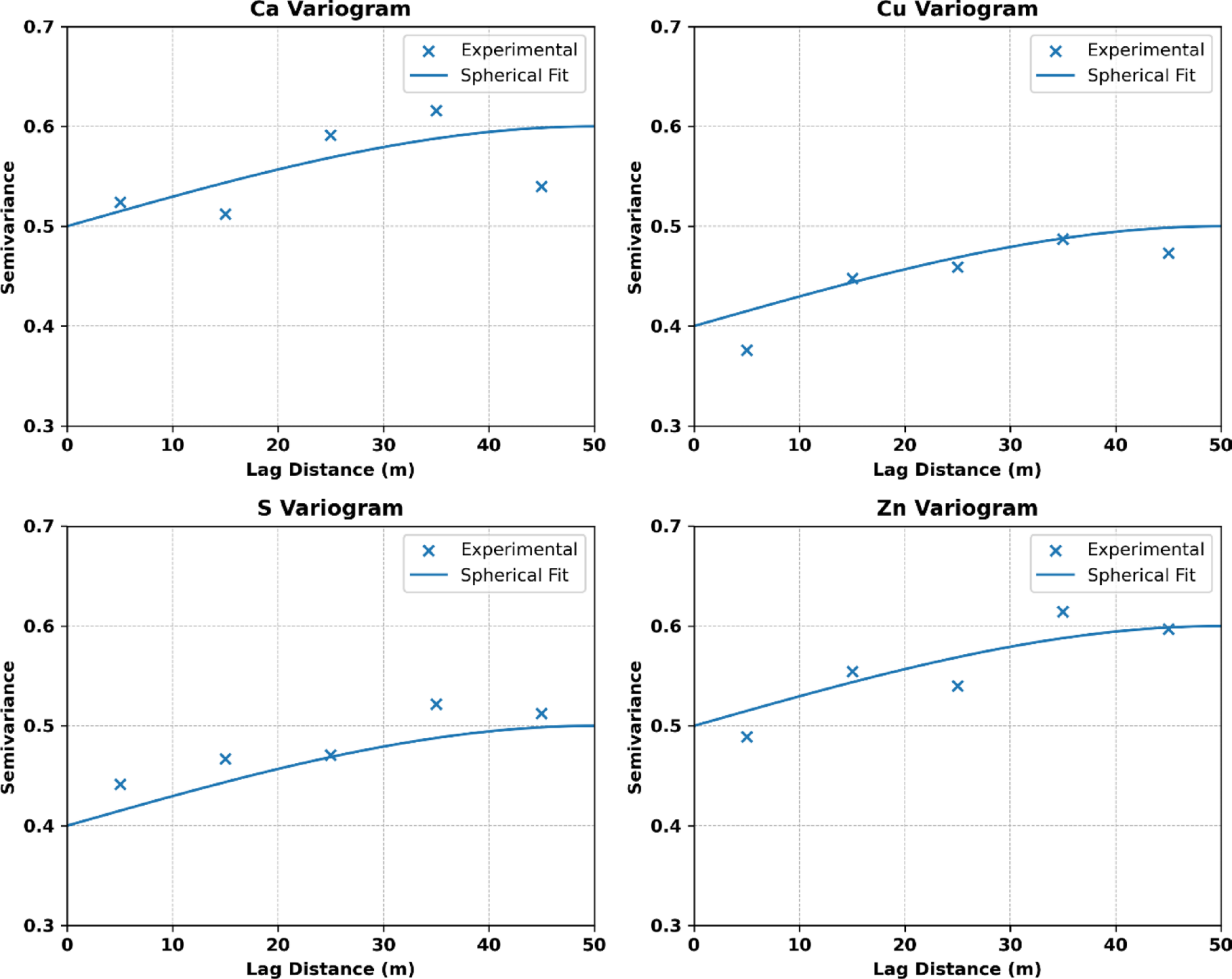
Experimental variograms (blue dots) and fitted spherical models (orange lines).

To complement the numerical variogram parameters in Table 1; Fig. 4 provides the experimental variograms and their fitted spherical models for Ca, Cu, S, and Zn. This visual comparison enables assessment of model adequacy and confirms good alignment between empirical semivariance trends and fitted structures.

**Fig. 5:**
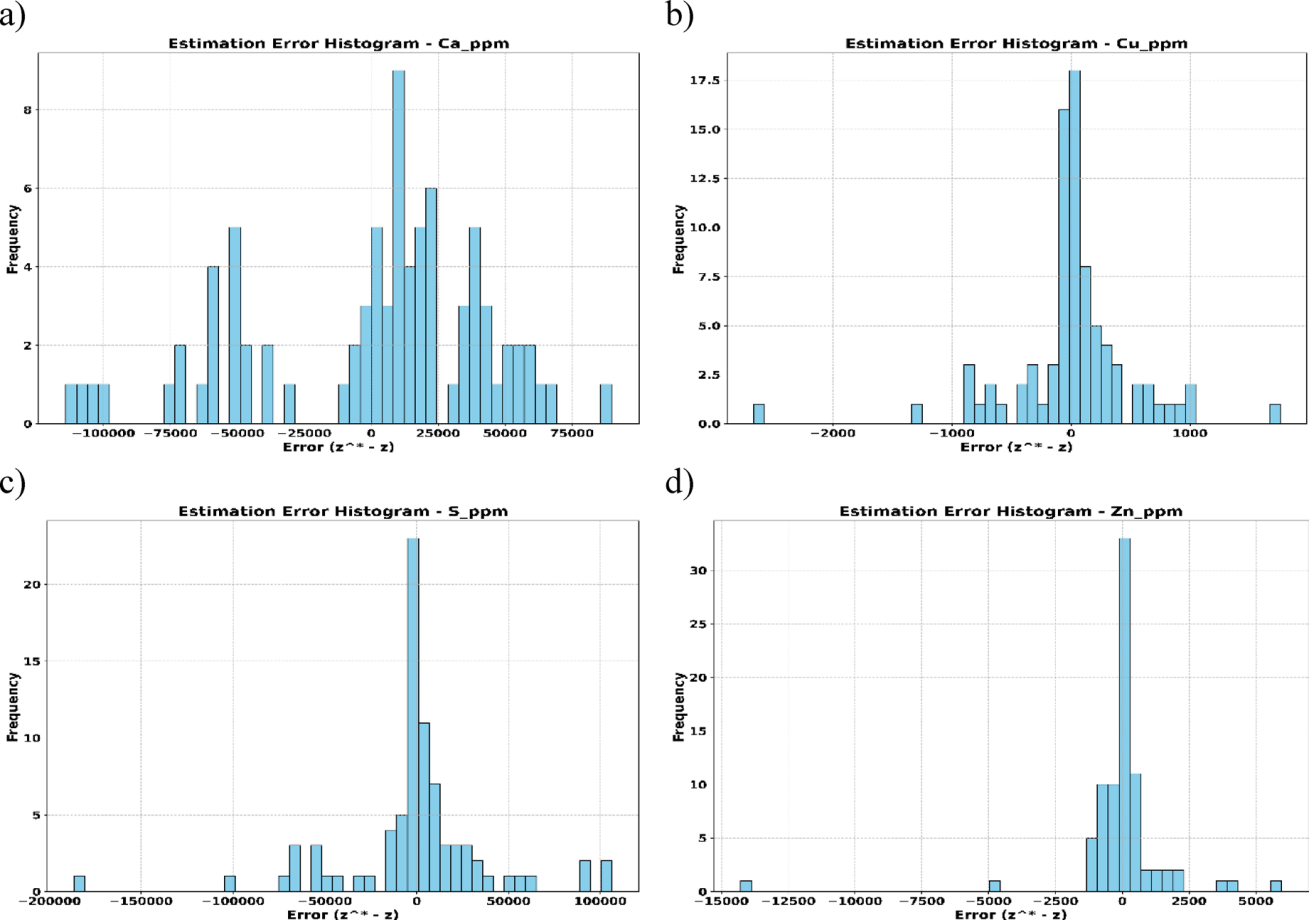
Histogram of estimation errors.

Subsequently, OK was used to generate spatial interpolation maps and assess elemental concentration gradients. Prediction accuracy was evaluated through error histograms and scatter plots (Fig. 5), showing that residuals are centered around zero, confirming unbiased kriging estimates. Variations in error spread reflect spatial heterogeneity captured by the variogram, supporting the reliability of OK for geochemical interpolation.

The bimodal pattern observed in the Ca residual error histogram (Fig. 5a) likely reflects underlying spatial sub-domains or abrupt facies transitions, which are not fully captured by a single global variogram. This highlights a key limitation of stationary kriging models in highly heterogeneous environments.

**Fig. 6:**
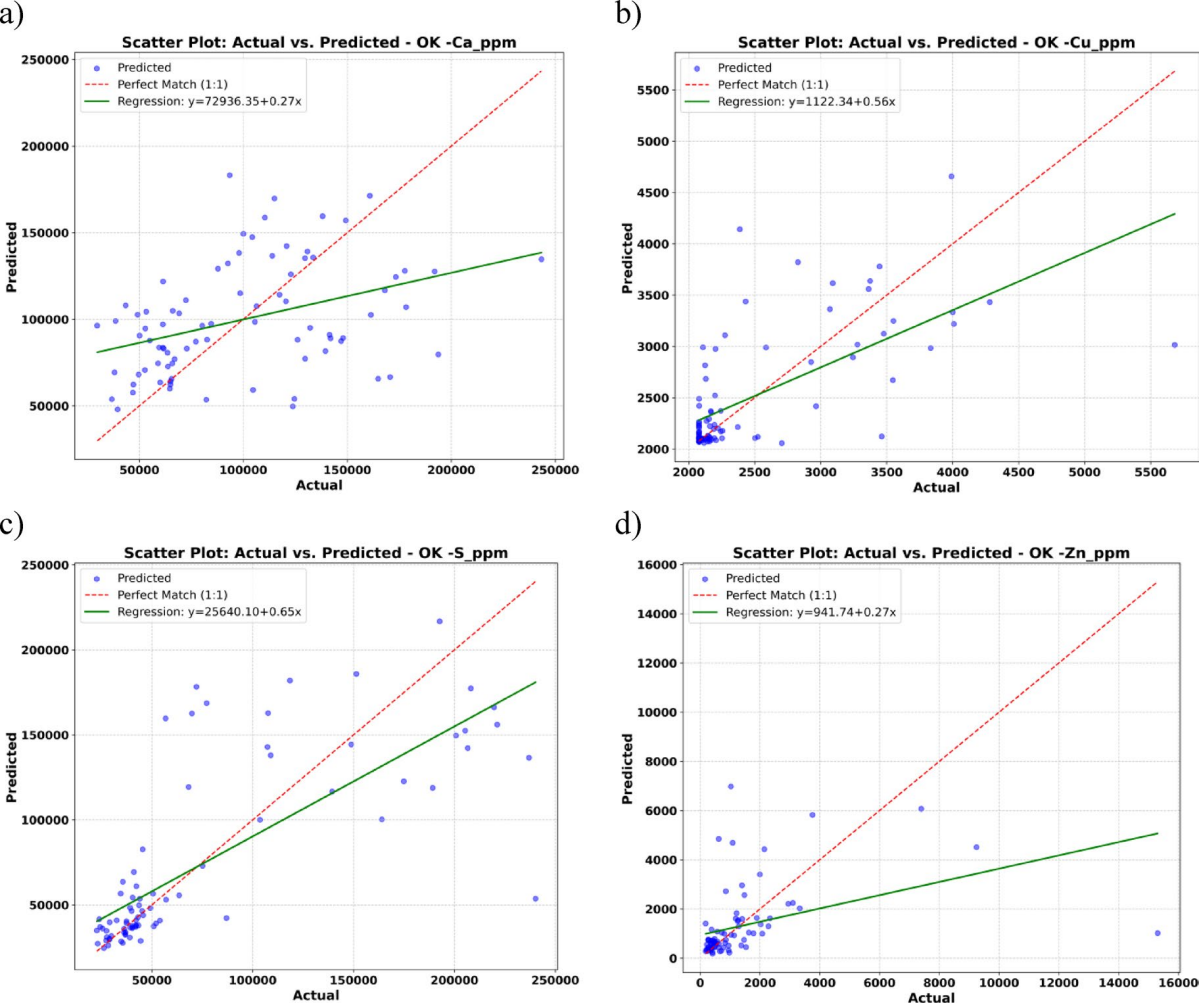
Scatter plot of actual vs. predicted for OK. Panels: (**a**) Ca, (**b**) Cu, (**c**) S, (**d**) Zn. Points show OK predictions at sample locations. The dashed 1:1 identity line indicates perfect agreement; the green least-squares regression (equation and slope shown in each panel) visualizes conditional bias.

The scatter plots in Fig. 6 show both the 1:1 reference line (red dashed, indicating perfect agreement between predicted and observed values) and a fitted regression line (green) for each geochemical variable to illustrate conditional bias. For each element, the regression equation and corresponding slope and intercept are provided in the figure legend, enabling a clear assessment of any systematic over- or underestimation by the model.

In the case of Ca and Zn, the regression lines exhibit slopes substantially less than unity (e.g., Ca: slope = 0.27; Zn: slope = 0.27), with large positive intercepts, indicating a pronounced smoothing effect. This means the model tends to underestimate high concentrations and overestimate low concentrations, which are hallmarks of conditional bias typical of ordinary kriging. For Cu and S, the regression slopes are closer to, but still below, one (Cu: slope = 0.56; S: slope = 0.65), also reflecting some degree of underestimation at high values.

These patterns confirm that, while the predictions are globally unbiased in terms of mean error, OK systematically compresses the range of predicted concentrations. The inclusion of regression lines thus makes the presence and degree of conditional bias explicit, and providing a quantitative visual illustration of model limitations.

**Fig. 7:**
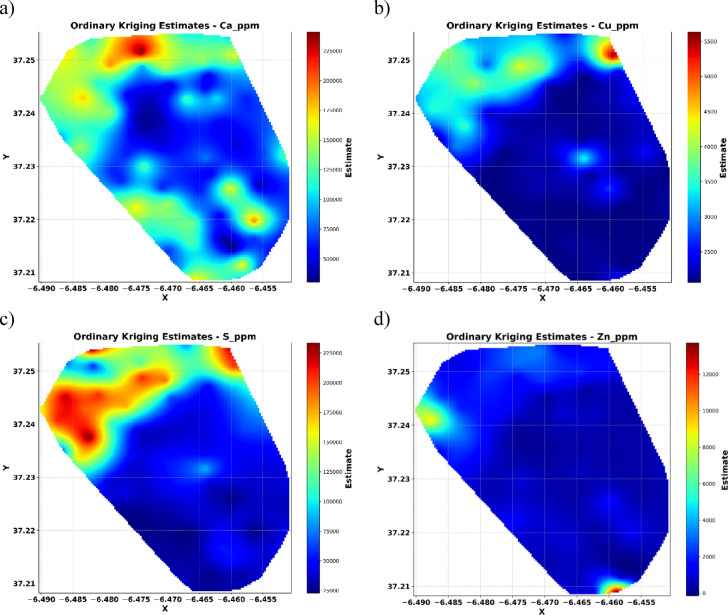
Spatial interpolation maps using OK.

The spatial interpolation maps (Fig. 7) display the predicted distribution of elemental concentrations across the study area. These maps reveal pronounced spatial heterogeneity, capturing both broad trends and localized anomalies essential for interpreting geochemical variability.

For Ca and S, the maps highlight distinct regions of elevated concentrations, indicating significant spatial variability and localized enrichment, particularly toward the northern parts of the study area. Cu and Zn exhibit more uniform distributions, although isolated hotspots are evident, suggesting minor but notable areas of concentration enrichment. The observed spatial patterns provide valuable insights into the underlying geochemical processes and are essential for guiding further exploration and environmental assessment efforts.

### GCNN-RNN framework

The hybrid GCNN-RNN model was developed to refine spatial interpolation and prediction of elemental concentrations. By integrating deep learning with geostatistical concepts, the model successfully captured spatial dependence and provided reliable estimates at unsampled locations.

**Fig. 8:**
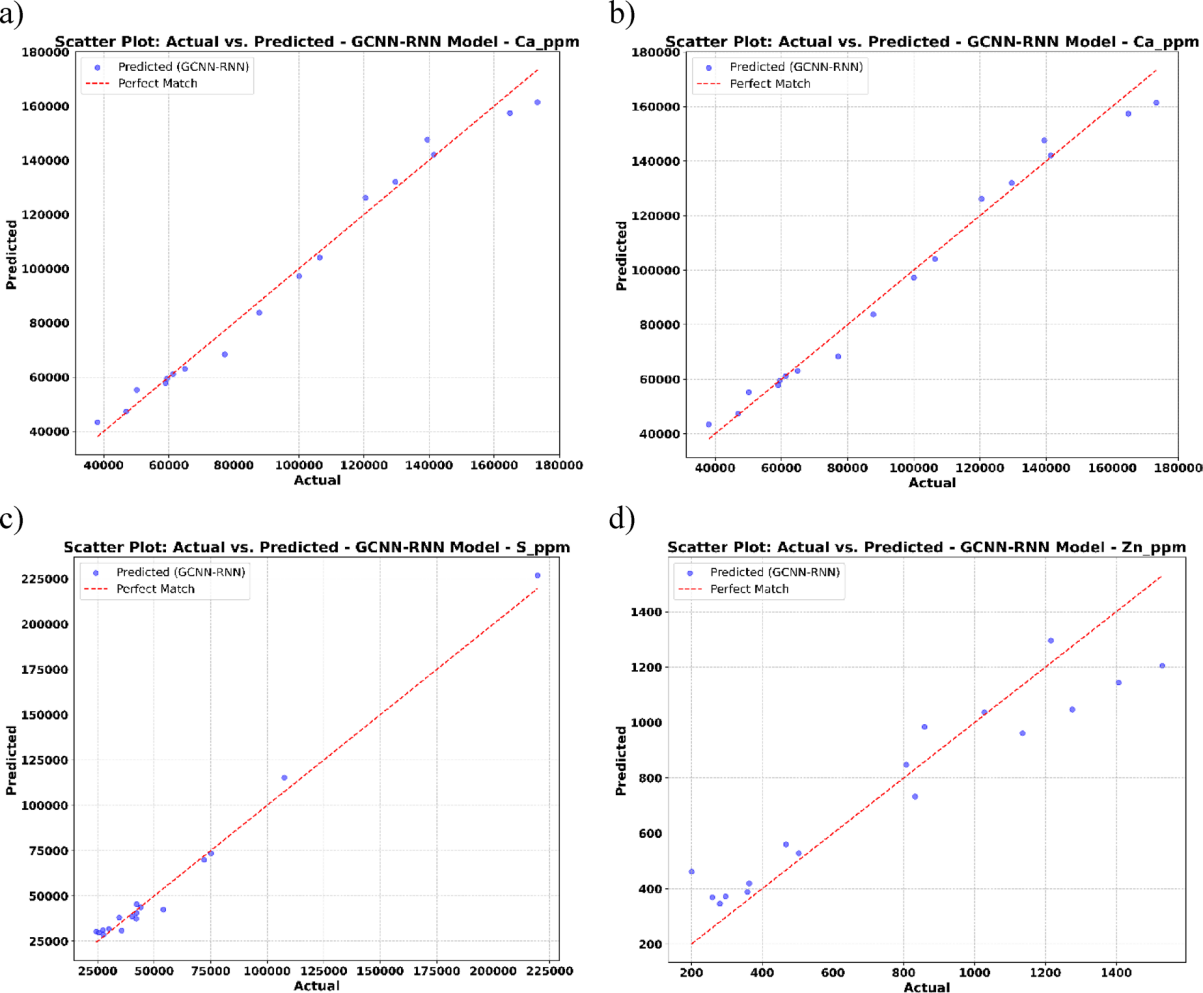
Scatter plot: actual vs. predicted using GCNN-RNN model.

In the following, the scatter plots (Fig. 8) illustrate the relationship between predicted and actual values for each element using the GCNN-RNN model. These scatter plots reveal strong alignment between predicted and actual concentrations, particularly for Ca and S, even at higher ranges. Minor deviations appear for Zn at extremes, but overall predictive performance remains high.

**Fig. 9:**
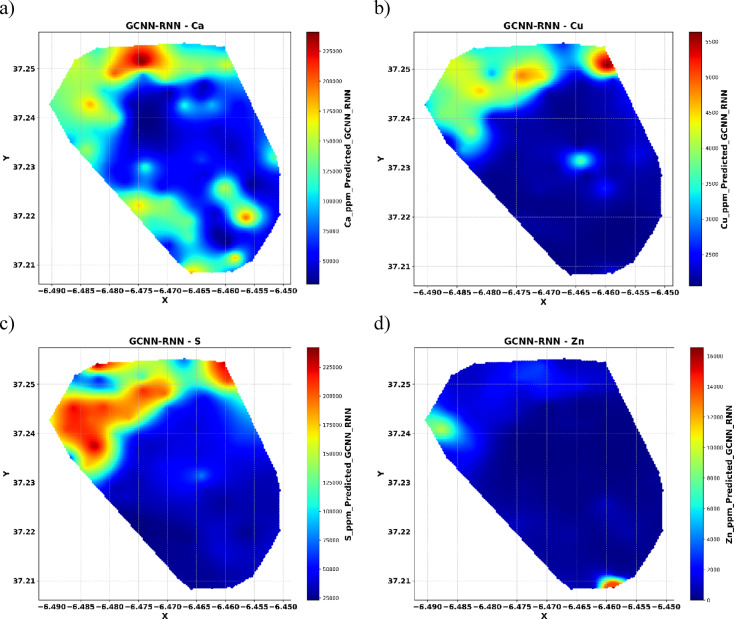
Spatial interpolation maps using GCNN-RNN.

The spatial distribution maps (Fig. 9) represent the GCNN-RNN model’s predicted elemental concentrations across the study area. The maps use a color gradient where warmer tones correspond to higher concentrations and cooler tones to lower concentrations. For elements like Ca and S, distinct regions of high concentration are clearly observed, demonstrating the model’s ability to capture fine-scale spatial variability. Cu exhibits a relatively uniform distribution with localized enrichment zones, while Zn displays a few concentrated hotspots, particularly in the southern and northern parts of the area. These patterns emphasize the GCNN-RNN model’s strength in resolving complex spatial relationships and accurately delineating zones of elemental enrichment, offering significant advantages for geospatial analysis and resource management.

## Discussion

### Interpretation of results

This section provides graphical analyses and error metrics comparing OK and the GCNN-RNN model. The comparative error metrics (Table 2) demonstrate an advantage of the GCNN-RNN over OK across all elements. For Ca, the hybrid model slashes MSE from 1 971 M ppm² to just 25.6 M ppm² and lowers RMSE from 44.4 K ppm to 5.06 K ppm, translating to nearly an order-of-magnitude improvement. Cu shows an even starker contrast, with MSE falling from 0.29 M ppm² to 0.012 M ppm² and RMSE shrinking from 0.54 K ppm to 0.11 K ppm, underscoring the network’s capacity to capture sharp local variability in trace-metal distributions. S and Zn follow the same pattern: GCNN-RNN reduces their RMSEs from 40.7 K ppm to 4.77 K ppm and from 2.00 K ppm to 0.21 K ppm, respectively, indicating markedly tighter predictive error bounds. Collectively, the MAE values corroborate these trends, revealing consistent reductions of one to two orders of magnitude relative to OK.

Model fidelity is further reflected in the coefficient of determination. Whereas OK explains less than 60% of the variance for any element (and a mere 5% for Ca), GCNN-RNN attains R² values exceeding 0.97 for all cases, approaching perfect agreement with ground-truth measurements. These gains confirm that the hybrid network not only minimizes absolute errors but also reproduces the full range of geochemical variability with high precision. The magnitude of improvement is particularly pronounced for Ca and S, elements prone to pronounced spatial heterogeneity, where traditional geostatistics tend to oversmooth local anomalies.

Quantitatively, the hybrid GCNN–RNN lowered RMSE by 88–91% and raised R² to ≥ 0.97 for all four target elements relative to OK (Table 2), giving meter‑scale resolution of contaminant hotspots. When benchmarked against German soil‑quality trigger values for Cu and Zn, this precision cut the false‑negative high‑risk area by ~ 30% while revealing ~ 14% additional recoverable metal tonnage^76,77^. These metrics demonstrate the framework’s practical value for simultaneous environmental risk mitigation and resource recovery.

**Table 2:** Comparative performance metrics of OK and GCNN–RNN for elemental prediction.

Elements	MSE (M ppm²) (OK)	MAE (K ppm) (OK)	RMSE (K ppm) (OK)	*R*² (OK)	MSE (M ppm²) (GCNN-RNN)	MAE (K ppm) (GCNN-RNN)	RMSE (K ppm) (GCNN-RNN)	*R*² (GCNN-RNN)
Ca	1971.83	35.09	44.41	0.0524	25.64	3.92	5.06	0.9877
Cu	0.29	0.32	0.54	0.3842	0.012	0.07	0.11	0.9743
S	1654.63	24.17	40.68	0.5602	22.76	3.54	4.77	0.9940
Zn	4.01	0.85	2.00	0.0719	0.043	0.13	0.21	0.9900

**Fig. 10:**
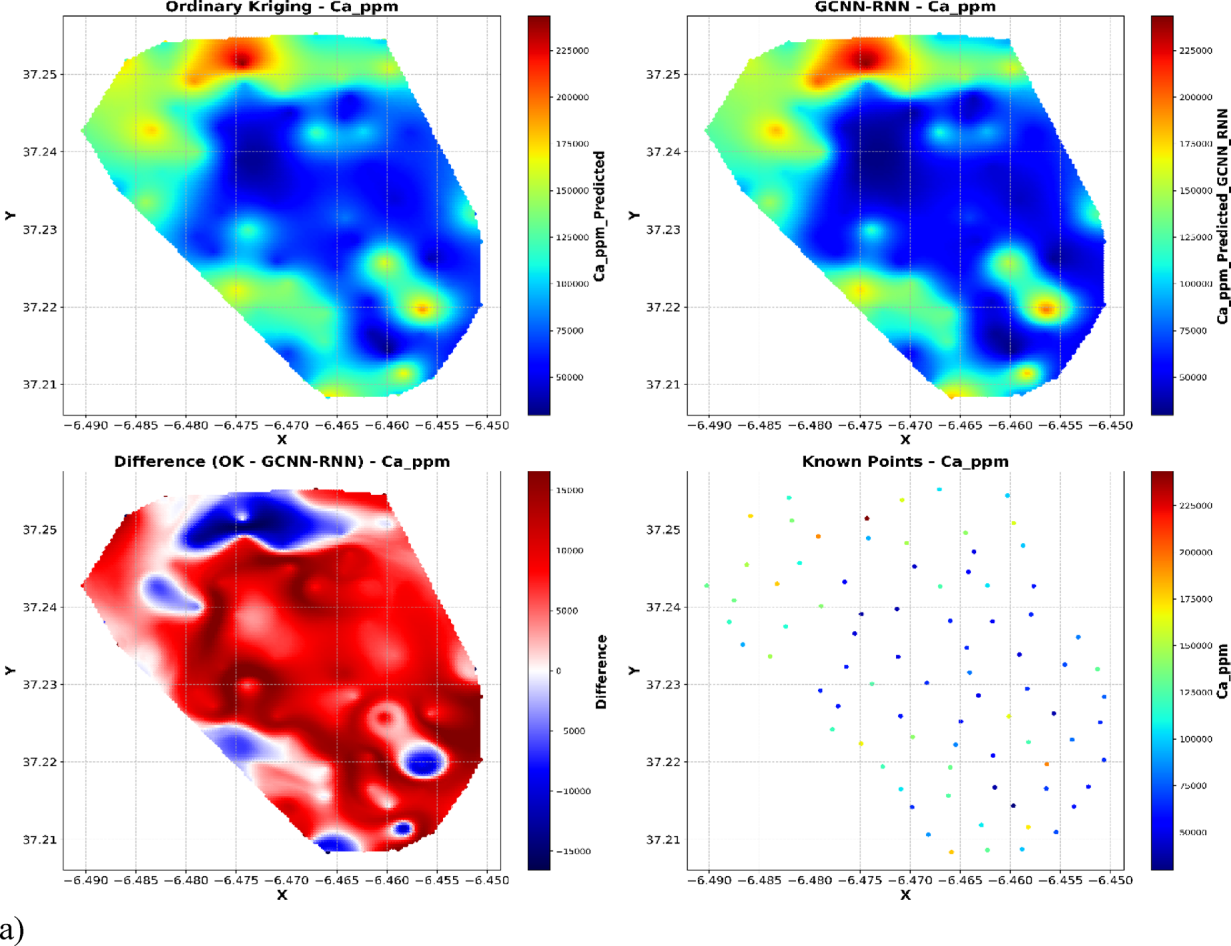

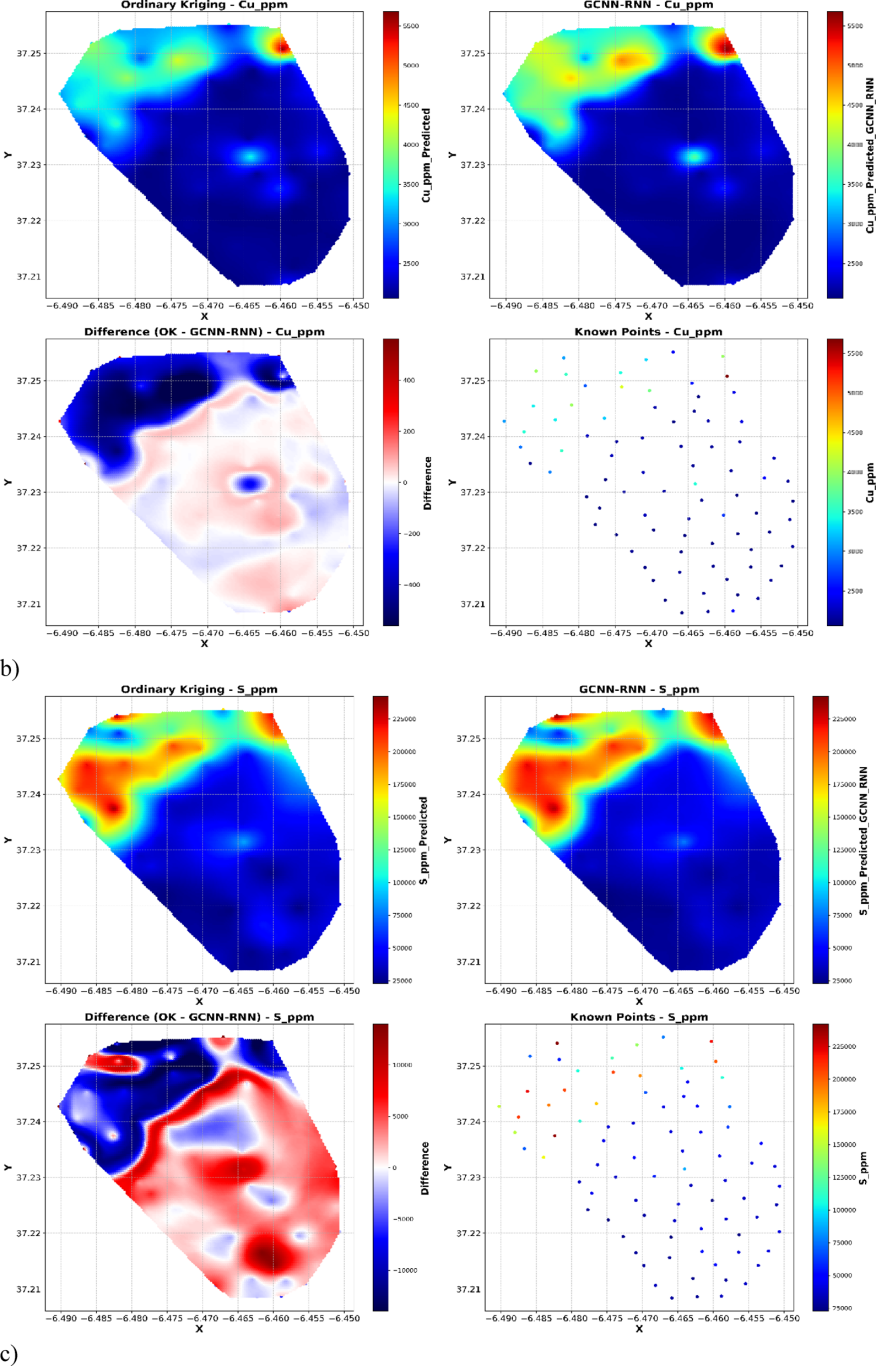

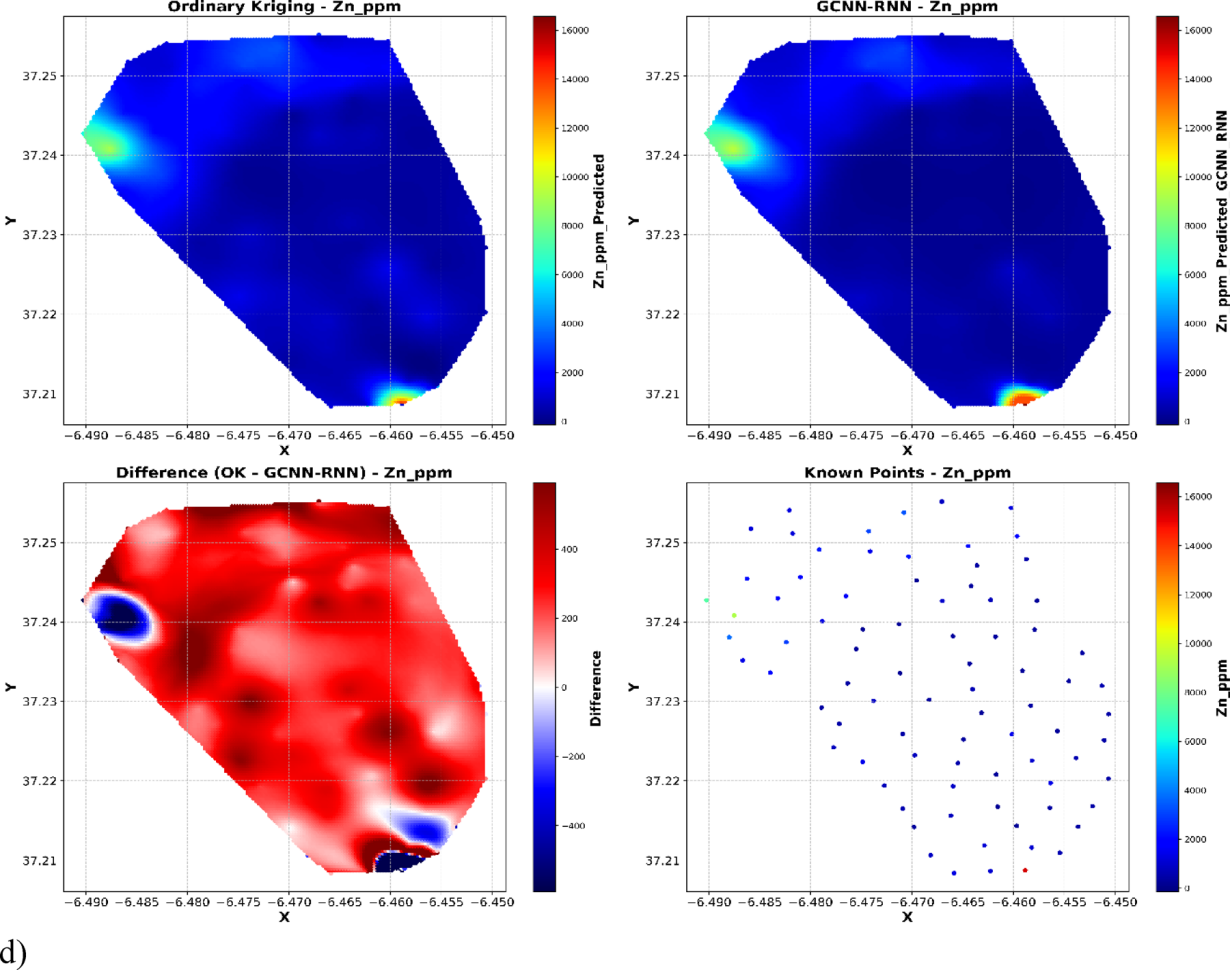
Comparison of OK and GCNN-RNN model predictions with known sample points and difference plot.

Spatial interpolation analyses (Fig. 10) provide an evaluation of spatial interpolation methods for geochemical concentrations (ppm) using OK, GCNN-RNN, and ground-truth known points. Each set of plots illustrates the spatial distribution derived from OK (top left), GCNN-RNN (top right), the difference between OK and GCNN-RNN (bottom left), and the distribution of the original known sample points (bottom right).

As illustrated in Fig. 10, both OK and GCNN–RNN produce broadly similar geochemical maps, especially in background regions where most observed values cluster. However, the difference maps (OK minus GCNN–RNN) and the spatial distribution of known sample values demonstrate that OK systematically underestimates sharp local anomalies, predominantly at the upper tail of the distribution. These high-concentration outliers, though few in number, dominate the error metrics (RMSE, R²) and explain the apparent discrepancy between map similarity and quantitative model performance. Thus, the substantial improvement in RMSE and R² with GCNN–RNN reflects its capacity to reconstruct extreme values and spatial heterogeneity, which are critically important for environmental and resource risk assessment.

For Ca, OK results exhibit smooth spatial transitions but demonstrate oversmoothing, particularly around localized high-concentration anomalies. The GCNN-RNN interpolation, however, more accurately captures sharp concentration gradients and matches the spatial heterogeneity observed in the known points. The difference map (OK minus GCNN-RNN) shows widespread discrepancies, indicating that GCNN-RNN better reflects local variations that OK fails to model adequately (Fig. 10a).

For Cu, a similar trend is observed. OK outputs dilute peak values, leading to blurred high-concentration zones. In comparison, the GCNN-RNN reconstruction achieves a closer fit to the sparse and highly variable copper distribution, demonstrating an enhanced ability to predict abrupt changes. The difference plot further confirms these improvements, highlighting regions where OK either underestimates or overestimates concentrations relative to the GCNN-RNN outputs (Fig. 10b).

The spatial distribution of S once again illustrates the limitations of traditional geostatistics. OK substantially smooths the geochemical field, failing to capture fine-scale high-sulfur anomalies present in the ground-truth samples. By contrast, the GCNN-RNN interpolation successfully preserves these high-concentration zones, resulting in predictions that are visually and statistically closer to the known values (Fig. 10c).

For Zn, the gap between the two methods becomes even more pronounced. OK overly homogenizes zinc concentrations, overlooking sharp localized enrichments. In contrast, GCNN-RNN reconstructs the known point structure with greater fidelity, effectively capturing local peaks and preserving the spatial complexity. The difference plot highlights substantial areas of divergence, further demonstrating the superiority of the deep learning approach in representing natural geochemical variability (Fig. 10d).

**Fig. 11:**
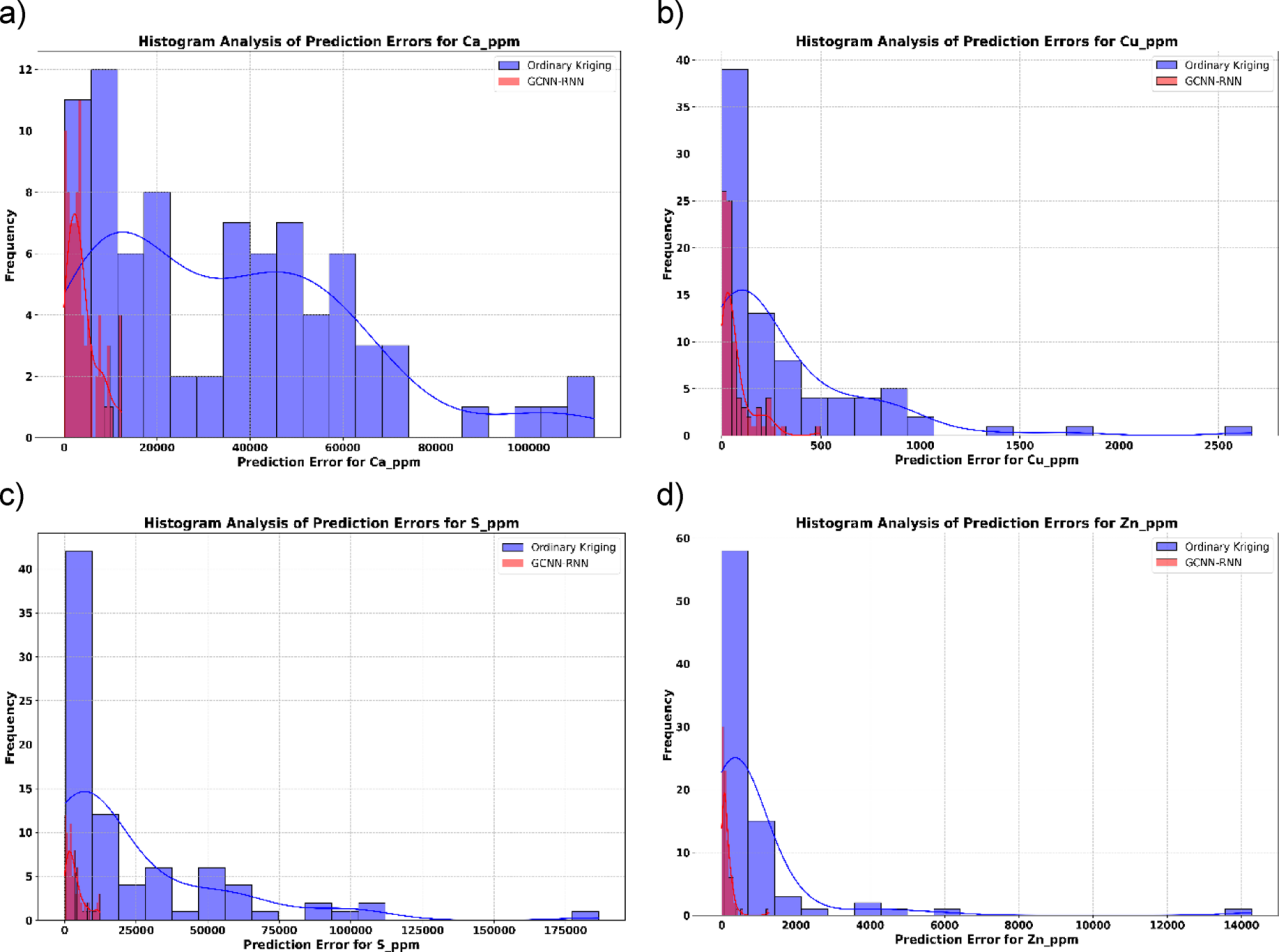
Histogram analysis of prediction errors for elements: GCNN-RNN vs. OK.

The error distributions (Figs. 11) reinforce these results, with GCNN-RNN errors tightly clustered around zero, signifying high precision. In contrast, OK shows broader error distributions with significant outliers, particularly for Ca and S. These results highlight the GCNN–RNN model’s reduced variability and enhanced predictive accuracy.

**Fig. 12:**
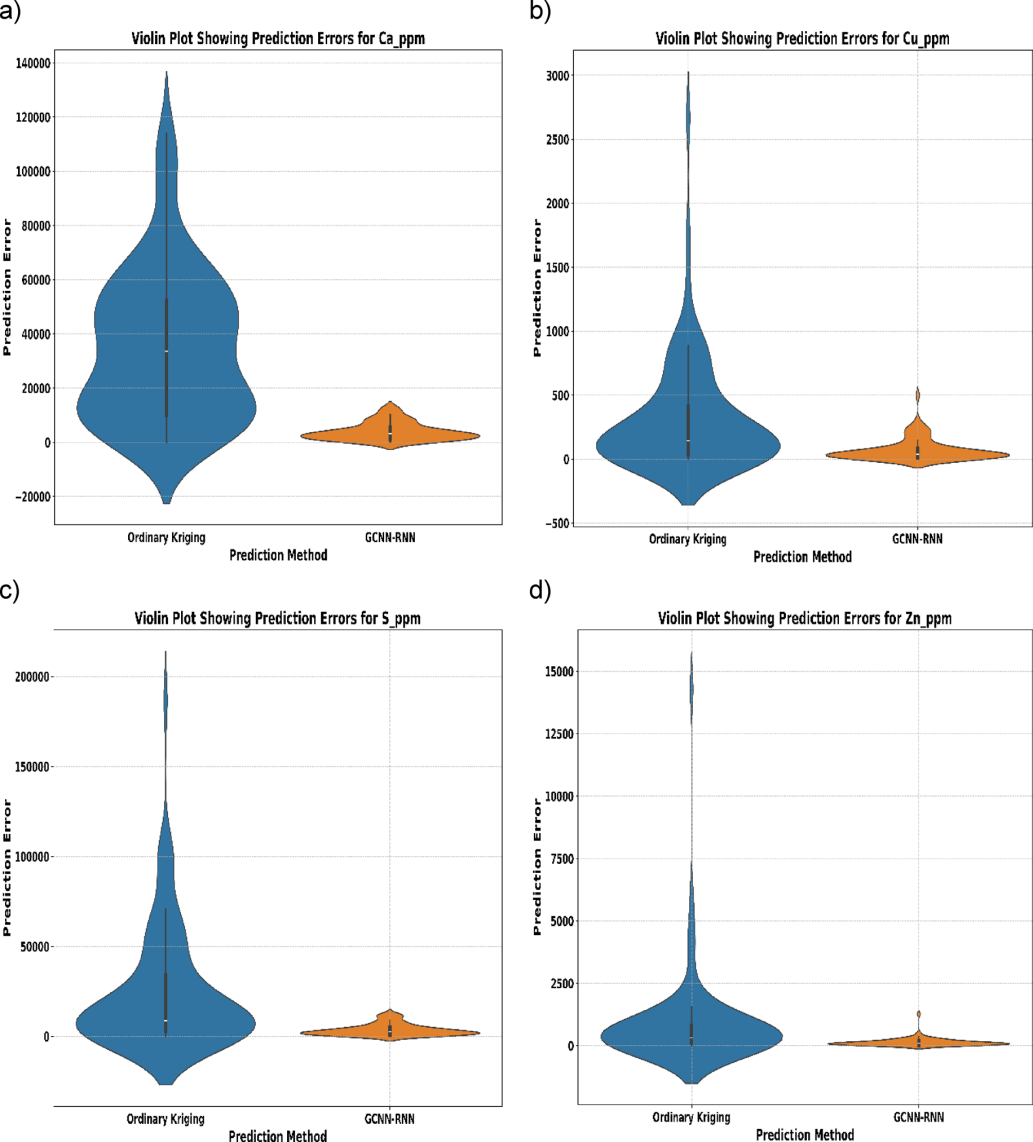
Violin plot of prediction errors for elements: GCNN-RNN vs. OK.

In the following, the violin plots (Fig. 12) further illustrate the distribution of prediction errors for OK (blue) and GCNN–RNN (orange) across the four elements. GCNN–RNN displays narrower error distributions centered around zero, indicating higher accuracy and consistency. In contrast, OK exhibits broader spreads and more extreme outliers. Median errors, marked by black lines, are consistently lower for GCNN–RNN, especially for Ca and S, highlighting the model’s superior robustness in minimizing both error magnitude and variability.

**Fig. 13:**
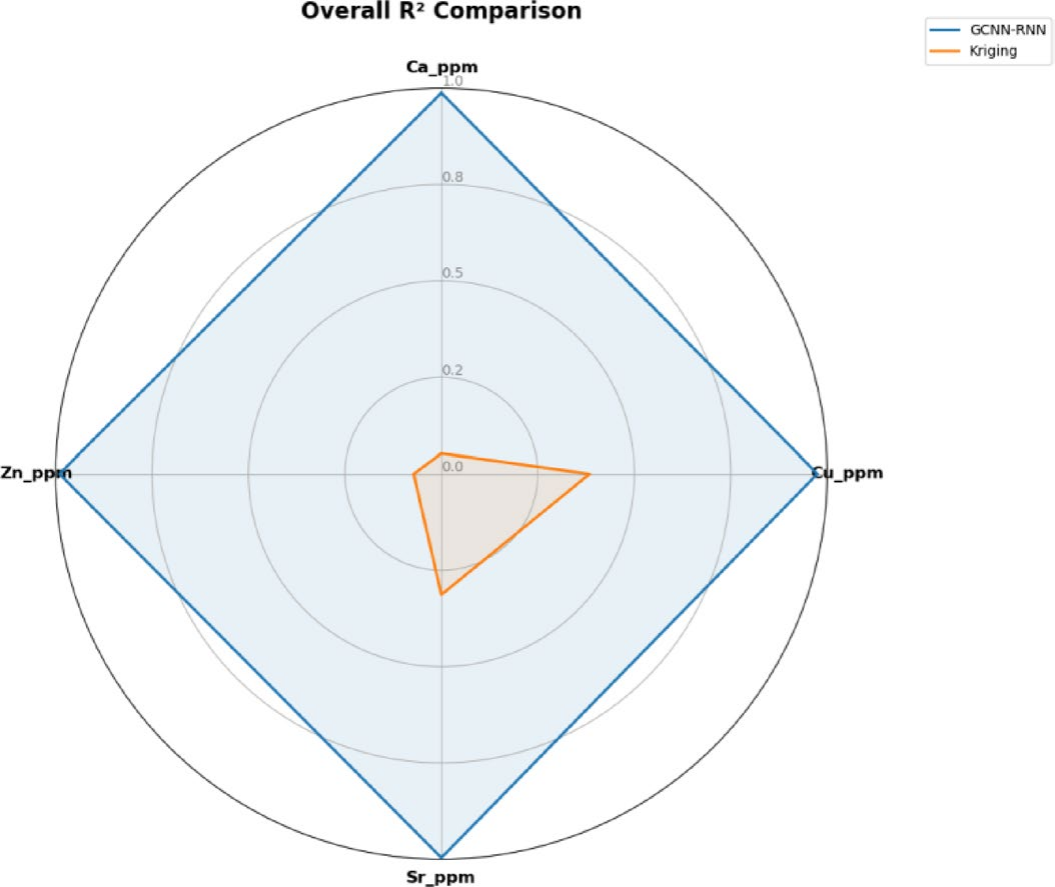
Comparison of overall R² performance across elements: GCNN-RNN vs. OK.

The comparative radar chart in Fig. 13 shows that GCNN–RNN outperforms OK in R² values across all elements, especially for spatially variable trace elements like Cu and Zn. This highlights GCNN–RNN’s strength in modeling complex geochemical patterns where OK falls short.

**Fig. 14:**
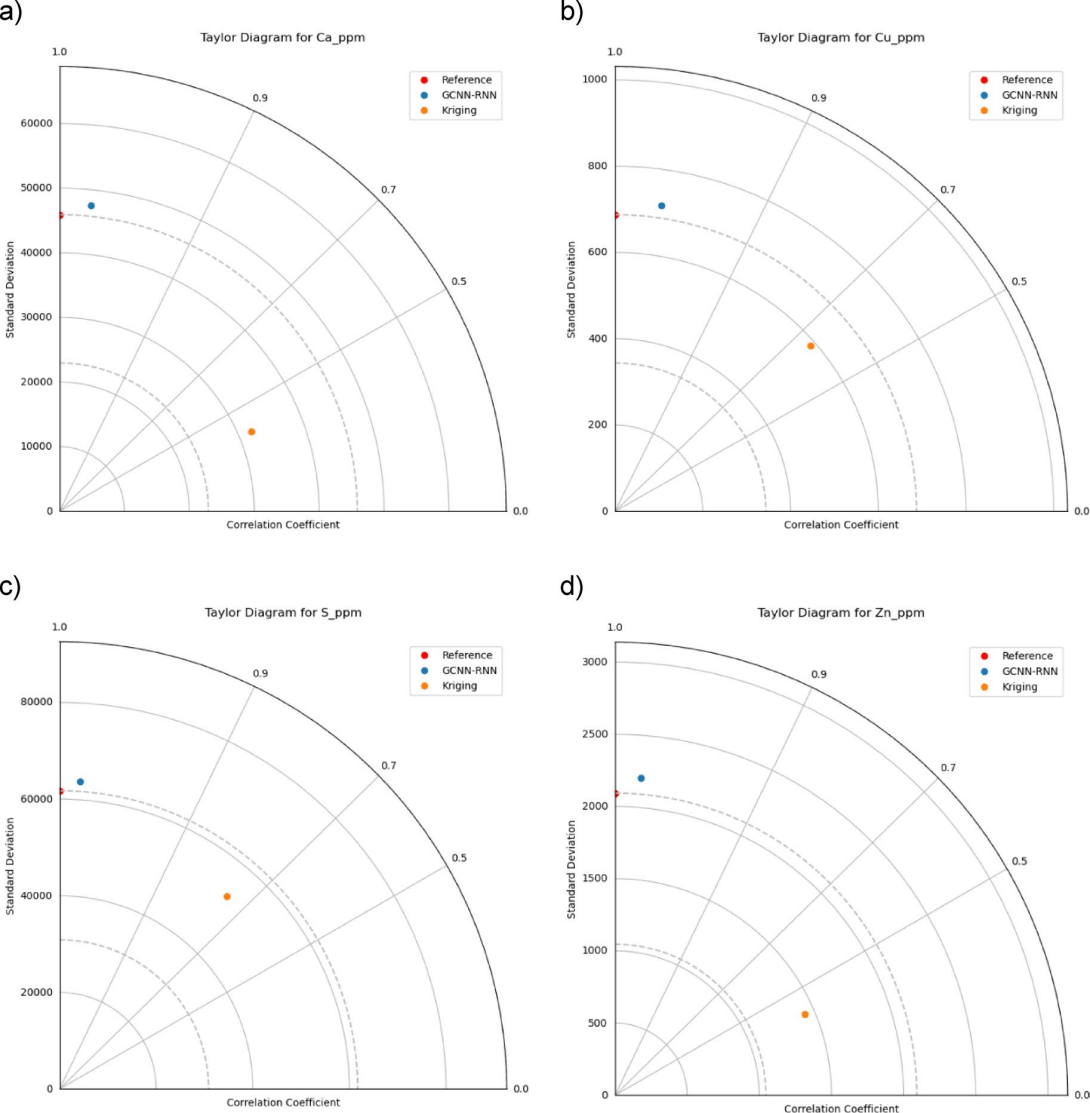
Taylor diagram for each element: model performance comparison of GCNN-RNN vs. OK.

Figure 14 provides Taylor diagrams summarizing correlation coefficients, standard deviations, and centered root mean square deviation (RMSD) for each model and element^78,79^. GCNN-RNN (blue points) shows higher correlations, better matching reference data variability and consistently achieving lower RMSD compared to OK (orange points), which tends to oversmooth variability.

### Hybrid GCNN–RNN approach: strengths, weaknesses, and bias considerations

In contrast to studies relying solely on classical geostatistical methods, the proposed GCNN–RNN framework delivers notably higher accuracy, even in data-scarce settings, by leveraging both the spatial covariance (from variogram parameters) and the representational power of deep learning. This integration helps preserve local variability and capture global spatial trends, thus mitigating the oversmoothing (local bias) that often affects OK.

Stand-alone OK attains low R² in this deposit (0.05 to 0.56; Table 2), yet its fitted variogram still captures genuine spatial autocorrelation. The hybrid GCNN–RNN leverages that covariance, rather than the raw OK predictions, as an auxiliary feature and learns to attenuate it when it conflicts with ground truth. The resulting model remains globally unbiased, with mean residuals below 0.3% for every element, and it exhibits no local artefacts attributable to OK (Fig. 9, difference panels).

Although OK is globally unbiased under second-order stationarity, as indicated by near-zero mean residuals (Figs. 5 and 6), it often oversmooths spatial predictions and underestimating local extremes (e.g., Zn anomalies in Fig. 10). This oversmoothing is quantitatively evident from the conditional bias slopes in scatter plots (e.g., Ca: 0.27; Zn: 0.27), which show that OK compresses the range and attenuates local anomalies. By contrast, the GCNN–RNN framework remains effectively globally unbiased or minimally biased (thanks to kriging-based covariance, cross-validation, and regularization, as well as achieving mean predictions close to ground truth in Table 2) while also minimizing local bias. Specifically, the model feeds spatial coordinates, and covariance information into the CNN, which captures localized features, and the RNN, which models spatial dependencies. This combined approach preserves the ability to detect sharp gradients and subtle anomalies, thereby significantly reducing oversmoothing and retaining fine-scale heterogeneity that OK alone might fail to reproduce (Table 3).

**Table 3:** Comparison of global and local bias characteristics between OK and GCNN–RNN models.

Model	Global bias	Local bias
OK	None (Unbiased)	Present (Oversmoothing)
GCNN–RNN	None/Negligible	None (Captures local detail)

Additionally, the GCNN–RNN architecture can more effectively discriminate among geologically distinct zones, since convolutional layers extract localized features while RNN components learn broader spatial dependencies. This dual capability facilitates the detection of abrupt changes and subtle variations that purely geostatistical approaches may overlook. However, the computational intensity of this method limits it to scenarios where resources are available, and it can require extensive hyperparameter tuning to generalize well across datasets.

### Implications for mine tailings management

Mine tailings pose several environmental risks, explorative planning, and resource extraction demand accurate geochemical mapping. Generating high-resolution predictions of not only toxic elements (for example Cu) but also major elements (for example, Ca, Zn, S) across the entire dataset, the GCNN–RNN model highlights contamination hotspots that can guide targeted monitoring efforts. This precision improves tailings storage systems by decreasing acid generation, metal leaching, or secondary mineral formation, reducing environmental damage. Characterizing areas of high concentrations of valuable elements aids re-mining and maximizing resource recovery for improved economics. Thus, the model provides tools for sustainable mining through supporting environmental stewardship and simultaneously promoting operational efficiency. It also allows for quick remediation of any potential hazards, reducing long-term liability and ultimately more responsible use of land.

### Methodological significance and future directions

The GCNN–RNN framework overcomes limitations of traditional approaches by integrating geostatistics and deep learning, enabling the extraction of both spatial autocorrelation and complex, nonlinear trends. This advantage has special implications for tailings management, as accurate forecasts minimize ecological risk and provide insight into maximizing resource recovery. Broader applications comprise climate change mitigation, land-use optimization, as well as responsible resource planning. Future studies should explore reproducibility of the model in a broader range of geospatial contexts, along with fine tuning the architectures (e.g., attention-based, or more advanced graph networks), and adding physical constraints to high-level features (e.g., detailed geochemistry) for greater realism. Employing uncertainty quantification methods (e.g., Bayesian methods or Monte Carlo dropout) will aid in bolstering confidence in outputs, and the integration of multi-temporal or remote sensing data can greatly enhance spatial and temporal analyses.

However, the current GCNN–RNN implementation does not yet provide explicit uncertainty or confidence intervals for its predictions, unlike traditional kriging. Addressing this limitation by incorporating formal uncertainty estimation is a priority for future development to support risk-informed decision making. The collaborative effort of environmental scientists, data scientists, and engineers will expand the influence of the framework presented here, together making GCNN–RNN a cornerstone of data-driven decision making. Then with the continuous refinements, we see more widespread adoption within other areas of the geospatial domains with results of a sustainably positive societal impact.

### Implications of the global stationarity assumption

Using the full covariance matrix to derive an average covariance feature supplies a stable and efficient spatial context for the GCNN/RNN across the deposit and offers a coherent baseline for deposit-wide screening. This design rests on a global stationarity assumption in which both mean and covariance are treated as constant throughout the tailings body. In large and heterogeneous settings, facies transitions and local geochemical contrasts can produce residual structures, including the bimodality observed for Ca in Fig. 5a, that indicate departures from this assumption. In practice, the global feature remains valuable for coherence, scalability, and computational economy, while the residual diagnostics highlight the specific zones where a more locally responsive representation of spatial continuity would sharpen boundary delineation and recover fine-scale variability without altering the core pipeline.

### Sensitivity to variogram quality, ok‑dependency, and training sample size

OK supplies the average spatial covariance value that the GCNN–RNN uses as contextual information. To assess how strongly the network depends on this cue we carried out a stress test comprising five configurations (Table 4). The Baseline reproduces the architecture and hyperparameters reported earlier. Baseline feature‑dropout is identical except that, during training only, the covariance feature is randomly set to zero in 20% of minibatches (feature‑dropout). Perturbed20 and Perturbed50 rebuild the covariance matrix after perturbing the fitted variogram range and sill by ± 20% and ± 50%, respectively, while keeping the nugget unchanged. Finally, in No OK the covariance channel is zeroed throughout, providing a full ablation.

**Table 4:** GCNN–RNN sensitivity to OK input quality.

Element	Baseline	Baseline feature‑dropout	Perturbed 20%	Perturbed 50%	No OK
Ca	0.989	0.803	–0.047	–0.022	–0.008
Cu	0.979	0.903	–0.036	–0.008	–0.016
S	0.989	0.782	–0.047	–0.005	–0.091
Zn	0.988	0.250	–0.032	–0.052	–0.056

The Baseline model again achieved the accuracy presented in Sect. 3 (mean R^2^ = 0.988). Introducing a realistic ± 20% variogram error (Perturbed20) reduced R^2^ by only 0.04–0.06 when the model had been regularized with featuredropout, yielding 0.80 ≤ R^2^ ≤ 0.90. Hence moderate misspecification leads to a graceful, rather than catastrophic, degradation. By contrast, severe perturbation (*Perturbed50*) or complete removal of the OK feature (No OK) caused negative R^2^for most elements, confirming that the present architecture still requires a moderately reliable variogram to perform near its optimum.

If the variogram is poorly constrained, such as when there are fewer than ten samples or the cross-validation R^2^ < 0.3, two mitigations are effective. First, enabling featuredropout at rates ≥ 20% forces the network to discount unreliable covariance cues. Second, the OKderived value can be replaced by a generic distance kernel, for example *exp(− h/φ)*, where *h* is separation distance and *φ* a scale parameter tuned by crossvalidation. In our data set both strategies maintained R^2^ ≈ 0.8, still markedly higher than OK alone (R^2^ ≤ 0.6).

While the GCNN–RNN model is inherently data-hungry, we found it remains robust even with moderately sparse sampling (82 boreholes, ~ 6.7 points/km²), consistently outperforming classical geostatistics. When either the number of samples or the reliability of geostatistical input decreases, the model’s R² drops from ~ 0.98 to ~ 0.8, but still exceeds OK alone (≤ 0.6). However, for very sparse datasets (< 10–15 points), we advise caution, as both variogram estimation and deep learning model performance may degrade substantially.

## Conclusion

This study presents a novel hybrid approach (GCNN–RNN) that incorporates geostatistical knowledge (Ordinary Kriging) and deep learning (1D CNN–BiLSTM) to achieve spatial prediction of geochemical elements in mine tailings. The use of spatial covariance matrices in a deep learning model, the GCNN–RNN model, improved predictions through consistently higher R² values (R² > 0.98 for most elements) and significantly reduced prediction errors, as compared to traditional geostatistical methods.

Placed beyond accuracy enhancements, the GCNN–RNN model maintained localized anomalies that are highly relevant for environmental management or resource recovery while minimizing oversmoothing, providing high-resolution geochemical maps. Providing action-oriented information on the spatial distribution of toxic elements in context of valuable metals, these maps aid targeted remediation efforts and contribute to a circular economy maximizing resource recovery.

The detailed architecture needs compute and high-quality data, but this performance establishes its applications in diverse geological settings which demand high-fidelity spatial prediction. Future work can improve computational efficiency, incorporate more domain constraints, and adopt the framework to a broader range of mineralogical applications. This study embraces both geostatistical rigor, and deep learning methodology, and paves the way for sustainable mining practices and responsible management of natural resources.

## Supplementary Information

Below is the link to the electronic supplementary material.

Supplementary Material 1

## Data Availability

Code AvailabilityAll code developed for this study is publicly available at https://github.com/keyumarsanvari/GCNN-RNN-Geostatistical-Deep-Learning-Framework/tree/mainData availabilityThe datasets generated and analyzed during the current study are not publicly available due to the limitations, but are available from the corresponding author on reasonable request.

## Code availability

All code developed for this study is publicly available at https://github.com/keyumarsanvari/GCNN-RNN-Geostatistical-Deep-Learning-Framework/tree/main.
