# Supplementary material for: Hybrid geostatistical and deep learning framework for geochemical characterization in historical mine tailings
Source: Sci Rep. 2025 Oct 7;15:35004. doi: 10.1038/s41598-025-19441-5 (PMC12504609; doi:10.1038/s41598-025-19441-5)
Supplement: Supplementary file 1 — Supplementary Material 1 [file 41598_2025_19441_MOESM1_ESM.pdf]

Supplementary Information

Hybrid Geostatistical and Deep Learning Framework for Geochemical Characterization in Historical Mine Tailings

This PDF accompanies the main manuscript and supplies the exploratory data analysis (EDA).

Supplementary Figure S1

Interpretation

- **Zn_ppm** and **Cu_ppm** exhibit right-skewed, heavy-tail distributions typical of trace metal assays; the occasional outliers (>10 000 ppm for Zn) justify the log-transform used later in modelling.
- **S_ppm** displays a pronounced bimodality, with a low-sulphur background (<50 000 ppm) and a high-sulphur mode (>150 000 ppm) corresponding to pyrite-rich horizons. This heterogeneity motivates our hybrid approach.
- **Ca_ppm** is multimodal and the broad tail towards >200 000 ppm reflects carbonate-rich layers that buffer acidity.

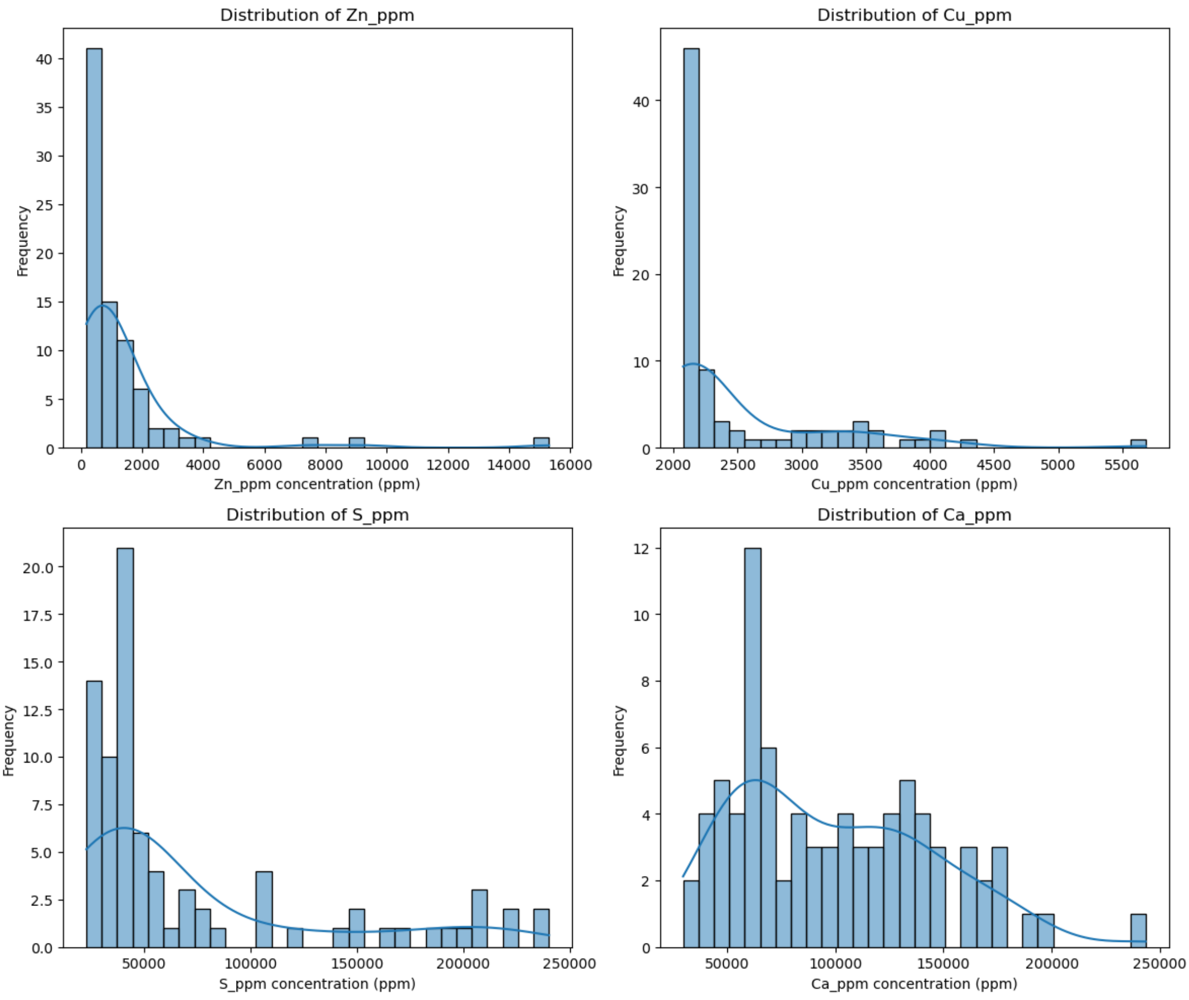

Supplementary Figure S1. Histograms and kernel-density estimates for the full synthetic dataset (Zn_ppm, Cu_ppm, S_ppm, Ca_ppm).

Supplementary Figure S2

Interpretation

- Medians (thick horizontal lines) and inter-quartile ranges (boxes) for all four elements differ by <5 % between the training and validation subsets, indicating that the random split preserved central tendency and spread.
- Whiskers and outliers extend to comparable maxima/minima in both subsets, ensuring that the validation fold challenges the models with the same extreme concentrations seen during training.
- This statistical balance eliminates sampling bias as a cause of performance differences between OK and GCNN–RNN.

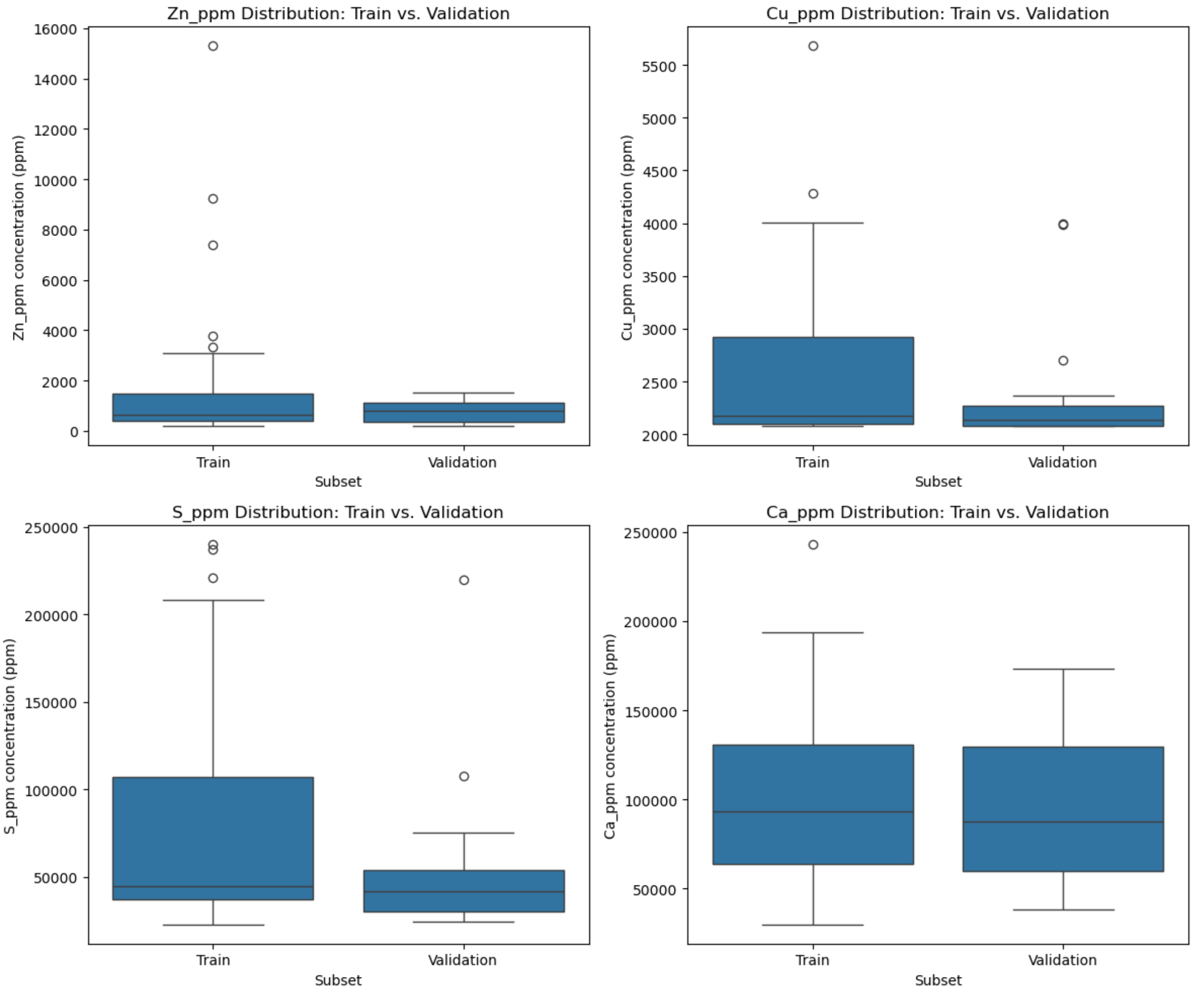

Supplementary Figure S2. Box-and-whisker comparison of training (80 %) and validation (20 %) subsets for Zn_ppm, Cu_ppm, S_ppm and Ca_ppm.
